# Electrochemical Atomic Force Microscopy of Black Phosphorus Composite Anodes: Electrode Destabilization and Degradation Mechanisms in Alkali-Ion Batteries

**DOI:** 10.1021/acsami.4c06693

**Published:** 2024-08-07

**Authors:** Samia Said, Rebecca R. C. Shutt, Zhenyu Zhang, Adam J. Lovett, Christopher A. Howard, Thomas S. Miller

**Affiliations:** † Electrochemical Innovation Lab, Department of Chemical Engineering, University College London, Torrington Place, London WC1E 7JE, U.K.; ‡ Department of Physics & Astronomy, University College London, Gower Street, London WC1E 6BT, U.K.; § The Faraday Institution, Quad One, Becquerel Avenue, Harwell Campus, Didcot, Oxfordshire OX11 ORA, U.K.; ∥ Renewable Energy Group, Department of Engineering, Faculty of Environment, Science and Economy, University of Exeter, Penryn Campus, Cornwall TR10 9FE, U.K.

**Keywords:** phosphorene, carbon, 2D nanomaterials, lithium-ion battery, EC-AFM

## Abstract

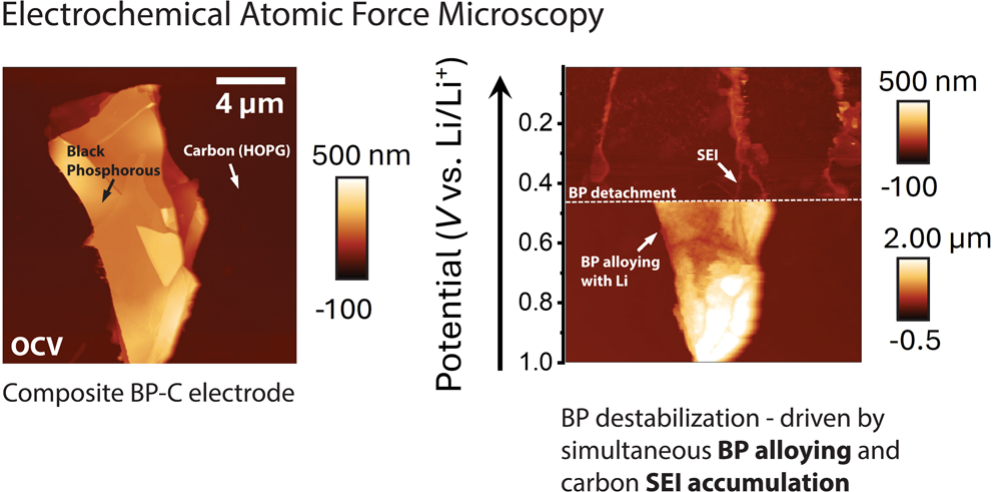

Despite their higher capacity compared to common intercalation- and conversion-type anodes, black phosphorus (BP) based anodes suffer from significant capacity fading attributed to the large volume expansion (∼300%) during lithiation. Downsizing BP into nanosheets has been proposed to mitigate this issue, and various methods, particularly mechanical mixing with graphitic materials (BP-C), have been explored to enhance electrochemical performance. However, the understanding of BP-C hybridization is hindered by the lack of studies focusing on fundamental degradation mechanisms within operational battery environments. Here we address this challenge by employing electrochemical atomic force microscopy (EC-AFM) to study the morphological and mechanical evolution of BP-C composite anodes during lithiation. The results reveal that BP-C binding interactions alone are insufficient to withstand the structural reorganization of BP during its alloying reaction with lithium. Furthermore, the study emphasizes the critical role of the solid electrolyte interphase (SEI) and BP-C interface evolution in determining the long-term performance of these composites, shedding light on the disparity in final electrode morphologies between binder-inclusive and binder-free BP-C composites. These findings provide crucial insights into the challenges associated with BP-based anodes and underscore the need for a deeper understanding of the dynamic behavior within operating cells for the development of stable and high-performance battery materials.

## Introduction

Elemental black phosphorus (BP) is an attractive anode material for lithium-ion batteries (LIBs)^1^ as like other known alloying materials such as Ge, Si, Sn, Pb, As and Sb, BP offers much higher volumetric and gravimetric energy densities than traditional graphite.^2,3^ This is because BP charge storage benefits from the intercalation of alkali ions and their alloying with the phosphorus host. This intercalation-alloying storage mechanism offers higher Li stoichiometries than traditional intercalation only electrodes, leading to a high theoretical specific capacity of 2596 mAh g^–1^ (Li_3_P), approximately 7 times larger than that of graphite (372 mAh g^–1^, LiC_6_).^4,5^

Unfortunately, batteries utilizing BP anodes suffer from significant capacity fade after only a few cycles. The alloying reaction is accompanied by a large volume expansion (∼300% for BP in LIBs),^6^ similar to that seen with conversion materials,^7^ which induces mechanical fracture and loss of electrical contact of the active material. It is widely accepted that large BP particles tend to be more fragile than smaller ones, resulting in lithiation-induced size-dependent fracture.^8^ Thus, reducing the size of BP particles, either by liquid phase exfoliation (LPE), or mechanical cleavage, has been suggested as an effective method in improving the electrochemical performance of BP.^9−12^ It is possible to suppress the volume expansion by limiting the lower anode voltage range to 0.78 V (vs Li^+^/Li).^13^ This prevents the reversible reaction between LiP and Li_3_P and thus enables a reversible specific capacity of around 600 mAh g^–1^. However, the resulting lowered energy density weakens the commercial appeal of P anodes.^13^

At present, a stable reversible capacity has not yet been achieved when accessing the full capacity of BP anodes.^14^ BP has been combined with several types of materials, including MXenes, metal–organic frameworks, and graphitic materials (graphene, carbon nanotubes, reduced graphene oxide) to control the volume change and to improve its electrical conductivity.^15−21^ Composite anodes mixed with graphite (BP-C) remains the most popular choice with several methods having been explored to form these hybrid anodes for alkali-ion batteries, each resulting in different structured electrodes.^22^ To date, the most widely adopted method for assembling BP-C hybrid composites is mechanical mixing as it is simple, scalable and can promote covalent bonding or van der Waals (vdW) interactions between BP and carbon. BP and graphitic materials have been combined in a solution or powdered form, using techniques such as low-energy ball milling, grinding, or stirring together,^13,23−30^ allowing composites to form via self-assembly during evaporation of the solvent.^31^ However, while mechanical mixing methods have shown promise, they often result in weak interactions between BP and the added materials. This limitation has been shown to hamper the synergistic effects necessary for stable battery performance.^12^ While high-energy ball milling has been employed to create BP-C composites with high initial capacities, a rapid capacity decay during extended cycling is still experienced in most cases.^26^ Although significant improvement in specific capacity and cycle life has been achieved through fabrication of different BP-C composites via mixing, a combination offering high initial Coulombic efficiency, high phosphorus loading,^32^ and high capacity with good stability at high rate of charge/discharge (>1 A g^–1^) is yet to be reported.

To improve the performance of BP-C anodes, a deeper fundamental understanding of the BP-C hybridization is required. However, studying this phenomenon is complicated by the fact that composite electrodes are widely used, which contain both the BP-C materials and polymeric binders (which are included to stabilize the electrode structure). Thus, it is non-trivial to deconvolute the benefits of layered material hybridization from the binder interactions. Specifically, understanding whether it is the polymeric binder or the carbon matrix that has the greatest impact on enhancing BP durability in composite electrodes. It is also noted that additional carbon is often added to electrodes to serve as a conductive additive, however, the impact of this material on electrode cyclability is often neglected. Consequently, there remains limited understanding regarding the specific role carbon plays in BP-C electrodes. While *ex situ* scanning electron microscopy (SEM) studies have been used to evaluate the morphological evolution of BP-C composite electrodes post-mortem,^23,33^ the spatial resolution of SEM is insufficient for assessing nanoscale structural changes. High-resolution transmission electron microscopy (TEM) has also been employed,^28^ however the post-cycling disassembly of cells, washing/drying of electrodes and exposure of materials to high vacuums and strong electron beams before imaging can induce unrepresentative electrode variations, or damage. Therefore, despite projections of mechanical failure such as cracking and pulverisation in BP-C composites, there is still limited understanding concerning the direct interrelation between BP and carbon, specifically in the context of their dynamic behavior within operating cells.

Our prior research on the sodiation of pure BP anodes is important to note here.^14^ With electrochemical atomic force microscopy (EC-AFM) we demonstrated that even when BP is downsized into nanosheets it remains susceptible to substantial structural alterations during sodiation due to alloying, including the breaking of the P–P bonds within the layered BP structure.^14^ Additionally, our investigations have shown that the solid electrolyte interphase (SEI) formed on BP in sodium ion batteries exhibits a problematic tendency to manifest as an inconsistent and unstable layer, contributing to the poor cycling performance of BP-based anodes. Collectively, these results would suggest that, due to the combination of pronounced structural reorganization in BP and the presence of unstable SEI layers, no level of hybridization could completely stabilize layered BP. Consequently, these results amplify the need to comprehend the stability of the BP-C structure in composite anodes for LIBs, plus the impact and role of SEI evolution at the BP-C interface on the electrochemical performance of BP-C anodes.

Herein, we present an EC-AFM study of the morphological and mechanical evolution of a BP-C composite anodes during lithiation under representative battery conditions. Our EC-AFM approach enables real-time investigation of evolving morphology and mechanical properties of the electrode/electrolyte interfaces while under electrochemical control.^34−38^ Our results demonstrate that the BP-C binding interactions alone are not sufficient to withstand the severe structural reorganization that BP experiences during its alloying reaction with lithium. These results provide significant insights into the crucial and incompatible role that both SEI formation on carbon and BP structural evolution due to alloying play in the poor long-term performance of these composites in real batteries. Additionally, our investigation emphasizes the disparity in final electrode morphologies between binder-inclusive and binder free BP-C composites, highlighting the primary role of the binder interactions over those of BP-C.

## Results and Discussion

### Electrochemical Characterization of BP-C Composites

**Figure 1:**
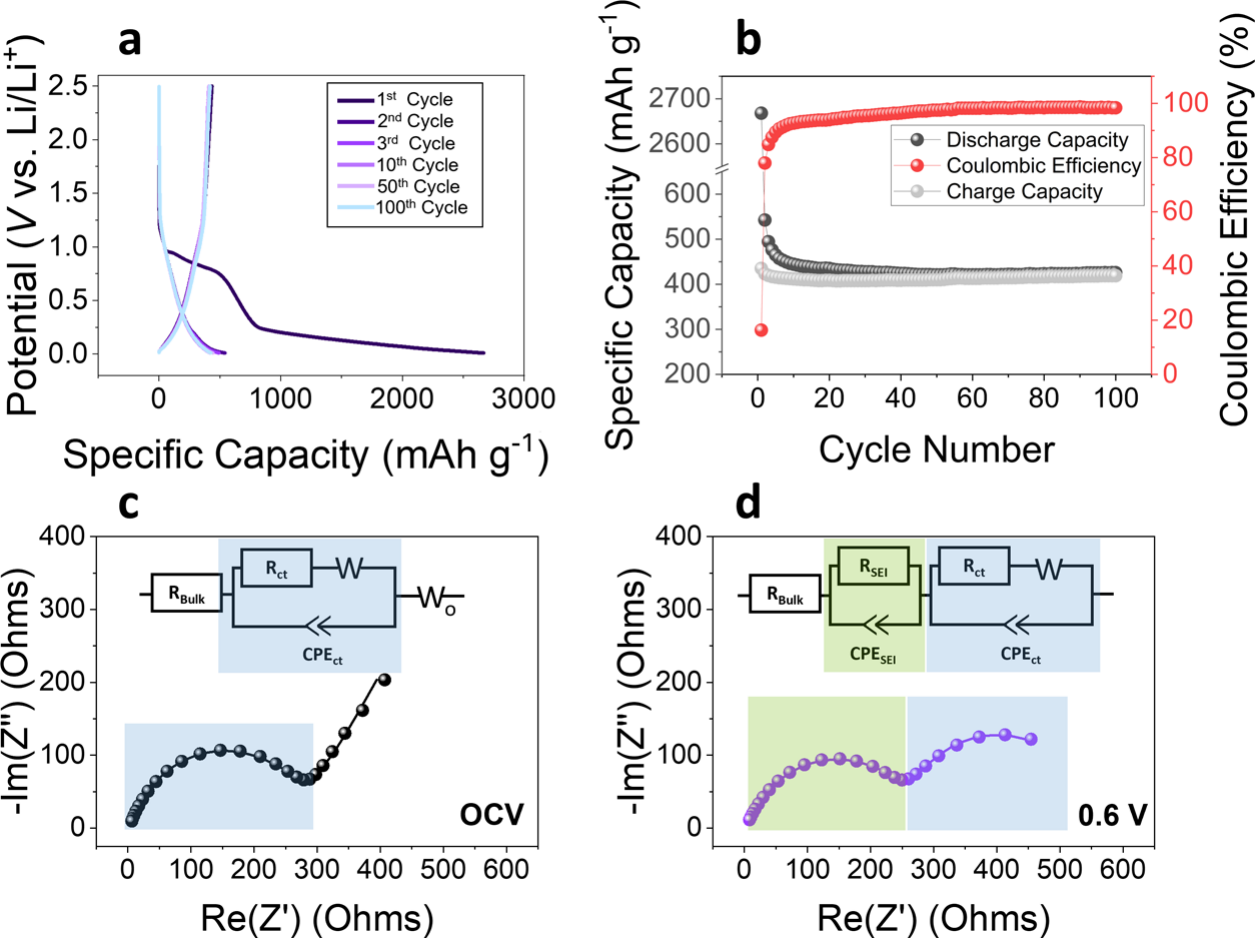
(a) Discharge–charge profiles of the binder inclusive BP-C(C45) composite electrodes during the 1st, 2nd, 3rd, 10th, 50th and 100th cycle between 2.5–0.02 V with a current density of 0.2 C (0.05 A g^–1^). (b) The reversible specific capacity and Coulombic efficiency of the BP-C(C45) electrodes vs. cycle number at 0.2 C. (c, d) Selected EIS Nyquist plots at (c) OCV and (d) 0.6 V after SEI formation measured in coin cells with BP-C(C45) anode vs Li metal. The full series is reported in Figure S1a,b. The circuit models used to fit the data are shown inset, with the fitted values reported in Figure S1c.

The electrochemical performance of binder inclusive BP-C (super C45) composite anodes (BP-C(C45)) was first investigated (Figure 1). Super C45 was chosen as the conductive carbon additive as it has been shown to be the most effective choice of carbon additive for electrode manufacturing, resulting in uniform electrode casts.^39^ Ultimately this translates to enhanced cell cycling performance of the electrode. For example, Si electrodes, which also suffer from volume expansion and pulverisation upon lithiation, have shown superior cycling stability with Super C45, compared to C65 and graphite.^39^ Super C45 is a graphitized form of carbon black^40^ that is known to lithiate (up to ∼250 mAhg^–1^) plus form SEI, consuming lithium in doing so.^41^ To account for this, the specific charge–discharge capacities were calculated accounting for the mass of BP and super C45 (i.e., the overall active mass).

Galvanostatic charge–discharge measurements of BP-C(C45) anodes were undertaken in coin cells vs Li metal in 1 M lithium hexafluorophosphate (LiPF_6_) ethylene/diethylene carbonate (EC/DEC) (1:1 vol %) electrolyte (Figure 1a,b). Cells were cycled between 2.5–0.01 V vs Li/Li^+^ at 0.2 C (0.05 A g^–1^). The charge–discharge profiles of the first, second, third, 10th, 50th and 100th cycles of the BP-C(C45) composite anodes are shown in Figure 1a. The first discharge cycle shows four distinct electrochemical processes. The first plateau between 1.2 and 0.9 V corresponds to the initial stages of BP-C SEI formation. The plateau between 0.9 and 0.75 V is likely to encompass additional contributions from initial stages of intercalation of lithium within BP layers, where the maximum theoretical intercalation capacity corresponds to Li_3_P_7_ (theoretical capacity 371 mAh g^–1^).^42^ The plateau at ∼0.7 V encompasses both faradaic processes in BP-C SEI formation and further lithiation of phosphorus up to stoichiometries of Li_2_P (theoretical capacity 1731 mAh g^–1^).^13^ Finally, the plateau at <0.2 V is related to the final stages of alloying, forming Li_3_P (theoretical capacity 2596 mAh g^–1^). In subsequent cycles, the plateau at ∼0.7 V is no longer present, as the phosphorene layers are not restored. Consequently, after the first cycle the discharge capacity decreased from 2667 to 543 mAh g^–1^ and gradually decreased to 427 mAh g^–1^ after the 100th cycle. This final capacity corresponds to less than one-sixth of the theoretical capacity of BP (assuming no contribution from C45), with the majority of BP degradation occurs in the early stages of cycle life.

The specific charge–discharge capacities and Coulombic efficiency over 100 cycles and are plotted in Figure 1b. The first cycle Coulombic efficiency is 16%. The low first cycle efficiency is attributed to a combination of SEI formation and BP-C detachment. This lowered first cycle efficiency could also imply that the degradation processes associated with BP lithiation, such as volume expansion and contraction during lithiation and delithiation cycles, might have compromised the covalent bonding/vdW interactions between BP and carbon. However, as the electrodes retain a stable capacity of ∼500 mAh g^–1^, after the second cycle (Figure 1a,b), this suggests that although the phosphorene layers were no longer restored, at least some of the phosphorus content had partially delithiated to Li_*x*_P (where 0 ≤ *x* < 3), or reformed to amorphous P. These results are consistent with the literature reports for BP-only LIBs,^13,43,44^ indicating that in this case, the performance of BP has not been improved by BP-C hybridization.

To further establish the relationship between electrochemical performance and electrode kinetics for BP in BP-C(C45) composites, electrochemical impedance spectroscopy (EIS) measurements were performed during the first charge–discharge cycle in coin cells (Figure S1a,b, Supporting Information). Selected EIS Nyquist plots of the BP-C(C45) composite anode at different states of charge are shown in Figure 1c,d, with the equivalent circuit model used inset. At OCV before SEI formation occurs (Figure 1c), the system is well-modeled by a Randles circuit in series with finite-length Warburg element (*W*_o_). The physical interpretation of this circuit is as follows: *R*_Bulk_ represents the ohmic resistance contributed primarily by the electrolyte plus contributions from the cell setup (current collectors, separator, cabling etc.); *R*_ct_ and CPE_ct_ correspond to the charge transfer resistance and double layer capacitance at the electrode/electrolyte interface, respectively. Note here a constant phase element was used to account for the electrode roughness and inhomogeneities in the system;^45^*Z*_w_ is the Warburg diffusion impedance of ions through the electrolyte; and *W*_o_ is attributed to the diffusion of lithium ions through a thin electrode^28^ and/or lithium ion diffusion via intercalation.^46,47^ After SEI formation (0.6 V, Figure 1d), the Nyquist plots exhibit an additional impedance arc at high frequency ranges (highlighted in green). It is well-modeled by the addition of an R-CPE element where *R*_SEI_ and CPE_SEI_ correspond to the resistance and double layer capacitance of the SEI layer, respectively.

The evolution of impedance during change and discharge is shown in Figure S1c (Supporting Information) with fitted values of *R*_ct_, *R*_SEI_ and *R*_total_ = *R*_Bulk_ + *R*_ct_ + *R*_SEI_ (vs charge–discharge potentialFigure S1c). Initially, during discharge to form lithiated-BP, *R*_ct_ falls from 270 Ω (at OCV) to 230 Ω (at 0.4 V). This trend is ascribed to the increasing metallicity of BP during lithiation as the lithium concentration (*x*) increases in Li_*x*_P, consistent with previous reports for BP electrodes.^14^ It is also noted that the below 0.6 V, there is no contribution from the finite length-open circuit terminus (*W*_o_). As *W*_o_ is related to the diffusion of ions through a thin electrode^28^ and/or the intercalation of Li through phosphorene layer,^46,47^ this suggests that below 0.6 V the BP-C electrode has significantly expanded, and/or lithium ions are no longer intercalating through phosphorene layers due to a change in the structure upon the formation of Li_*x*_P. These results are consistent with the galvanostatic cycling data presented in Figure 1a,b, which shows significant electrochemical activity around ∼0.7 V from the amorphization of BP though lithiation of stoichiometries up to Li_2_P.^13,43,48,49^

Further lithiation down to 0.01 V resulted in an increase in *R*_ct_ and a decrease in *R*_SEI_ (Figure S1c, Supporting Information). This is primarily attributed to active material isolation caused by significant volume change of electrode material, resulting in a more resistive charge transfer process. In turn, this volume expansion caused the destruction and dissolution of the SEI layer on the BP-C(C45) electrode surface, which reduced its resistance, as has previously been reported from EIS studies of MoS_2_ electrodes.^50^ Upon subsequent delithiation during charging, no significant change in *R*_SEI_ and *R*_ct_ was observed until 1.5 V, where *R*_total_ sharply decreased. This could indicate further partial removal of the SEI at the surface of the electrode, therefore reducing the contribution of *R*_SEI_, resulting in a lower *R*_total_. But by 2.5 V (Figure S1c) *R*_total_ increases again, with a significant net-increase compared with OCV (270 Ω at OCV vs 328 Ω at 2.5 V post first cycle). This increased resistance results from a combination of the electrical isolation of active material, the partial reformation of the highly resistive BP, and SEI formation/dissolution.

**Figure 2:**
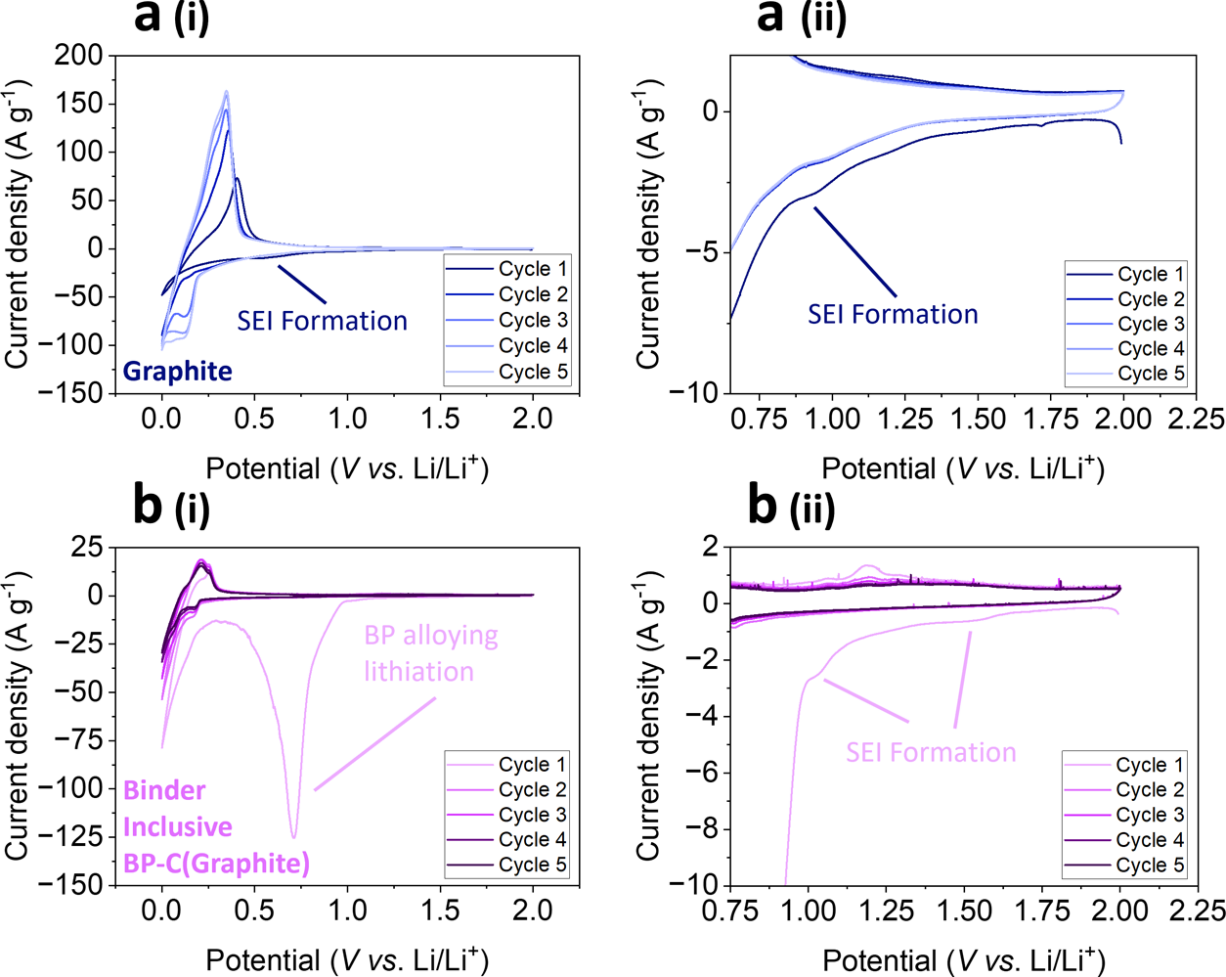
Cyclic voltammograms of (a) binder inclusive graphite reference and (b) binder inclusive BP-C(graphite) half cells vs Li/Li^+^. SEI redox peaks are labeled for both electrode compositions in (ii). All cells were cycled 5 times at 0.2 mV s^–1^ with the electrolyte 1 M LiPF_6_ EC/DMC in coin cells.

While the origins of the electrochemical signatures discussed above are widely reported,^23,47,51−53^ few studies directly link the electrochemistry to physicochemical change via *in situ* or *operando* experiments. This means the intricacies and interconnections between electrochemical, morphological and compositional change are largely unexplored. This warrants our EC-AFM approach, which is discussed in the following sections. Prior to EC-AFM experiments, the electrochemical performance of binder inclusive BP-graphite composite anodes (BP-C(graphite)) was evaluated with cyclic voltammetry (CV). Here, super C45 was replaced with graphite as the conductive carbon to replicate CVs generated from the EC-AFM (which utilize HOPG due to its atomic flatness which maximizes resolution). Figure 2 reports CV curves of the first five cycles for the binder inclusive graphite reference (Figure 2a), and BP-C(graphite) (Figure 2b) electrodes vs Li metal in 1 M LiPF_6_ in ethylene/dimethylene carbonate (EC/DMC) (1:1 vol %) electrolyte performed at a scan rate of 0.2 mV s^–1^. Figure 2a(ii),b(ii) show the magnified current responses in the early stages of the cathodic polarization for both electrodes.

The CV of the graphite electrode (Figure 2a) shows limited reductive current associated with lithium intercalation (<0.3 V) on the first cathodic scan. However, a broad peak between voltages 1.4–1.0 V is observed that is not present in subsequent cycles and is attributed to SEI formation (labeled). In cycles 2–5, three reduction peaks developed between 0.2 and 0.08 V, 0.08 and 0.02 V, and close to 0.001 V, assigned to lithium intercalation of the graphite. These are reversible with corresponding oxidation peaks on the anodic scan at ∼0.3 V and 0.35–0.45 V. The overpotential for both the reduction and oxidation peaks for lithiation of graphite reduces as the cycle number increases.

During the first cathodic polarization of the BP-C(graphite) composite (Figure 2b), the onset potential, calculated from interpolating the current back to zero, is measured to be approximately 2.0 V. The first reduction peak on the cathodic scan occurs between 1.65 and 1.4 V. The second reduction peak occurs between 1.1 and 1.0 V (labeled). These current responses have been attributed primarily to the irreversible decomposition of the electrolyte to form an SEI layer on the surface of the BP-C(graphite) anodes, which has been reported to occur between 2.0–0.6 V for BP^14^ and between 1.75–0.4 V for graphite^54,55^ in the same electrolyte composition (1 M LiPF_6_ in EC/DMC, 1:1 vol %). Further cathodic polarization results in the growth of a broad feature between 1.0 and 0.5 V, with a maxima at 0.65 V and a shoulder at ∼0.9 V. From the voltage ranges, the broad peak can be attributed to both faradaic processes in SEI formation and lithiation of phosphorus up to stoichiometries of Li_2_P.^13,43,48,49^ A final reduction peak at voltages close to 0.01 V is attributed to both the lithiation of graphite and the formation of Li_3_P. On the anodic scan, an oxidation peak ∼0.3 V from the delithiation of graphite is observed. Additionally, only one slight oxidation peak was observed at ∼1.25 V in the first cycle of the BP-C(graphite) composite, possibly corresponding to the partial delithiation of the phosphorus. However, due to the lack of electrochemical activity observed in subsequent cycles, the lithiation of phosphorus is assumed to be irreversibly forming Li_3_P which becomes electrically isolated. These results are consistent with the data presented in Figure 1, confirming that the carbon component (either super C45 or graphite) in the binder inclusive BP-C composites was not able to effectively mitigate or endure the degradation processes associated with BP. Consequently, this leads to persistent changes in the electrochemical behavior of the composite electrodes in subsequent cycles. This observation is crucial for understanding the stability and long-term performance of BP-C composites in LIB applications.

### EC-AFM Imaging of the BP-C Lithiation Mechanism

**Figure 3:**
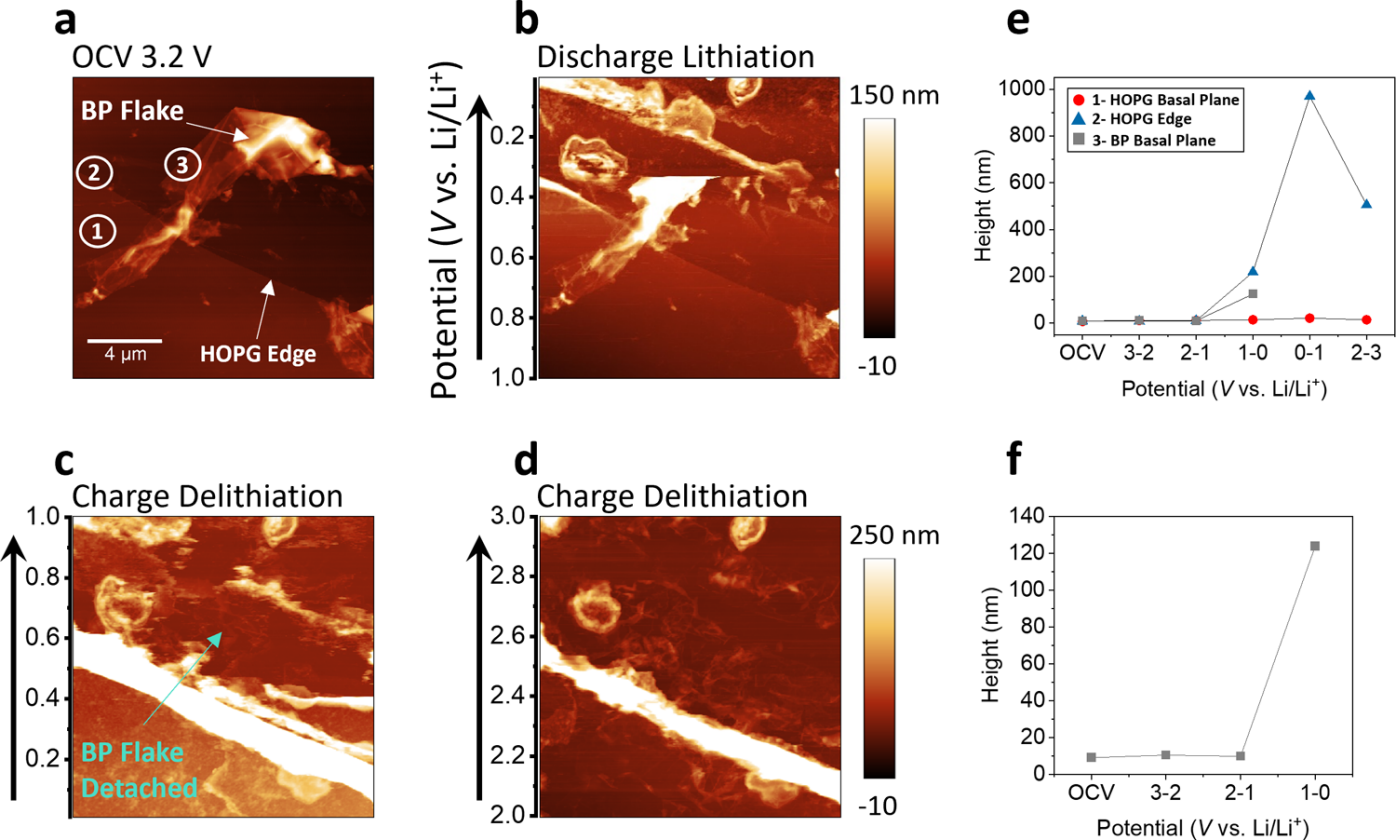
EC-AFM images of a binder free BP-C(HOPG) anode collected while under electrochemical control in the range 3–0.01 V at 0.5 mV s^–1^ in 1 M LiPF_6_ EC/DEC electrolyte vs Li/Li^+^. Selected EC-AFM images: (a) at OCV, (b) 1.0–0.01 V during discharge, (c) 0.01–1.0 V during charge (d) 2.0–3.0 V during charge. The full series is reported in Figure S7, Supporting Information. All EC-AFM images are captured across a 15 × 15 μm^2^ scan area, voltages plotted vs Li/Li^+^, with the scale bar inset in (a). (e, f) Relative height changes from a HOPG basal plane, HOPG step edge, and BP basal plane (labeled 1, 2, and 3 respectively in (a)) plotted vs cell potential window (the voltage range of the AFM image). The height change of the BP basal plane is plotted separately in (f) for clarity.

It has been established above that while much capacity of binder inclusive BP-C composite anodes was lost during repeated charge–discharge cycles, the electrodes maintained a partial reversible capacity of ∼400 mAh g^–1^ (up to 100 cycles). However, given that in these electrode architectures BP is combined with both conductive carbon and binder, it is impossible to decouple the influence of BP-C interface, from that of the binder-(BP-C) interaction. Therefore, to establish to the degree of benefit to the performance from BP-C covalent bonding/vdW interactions alone, binder free BP-C composite electrodes were prepared and investigated with EC-AFM during the first cycle of operation (Figure 3). The binder free BP-C electrodes were prepared by drop-casting BP flakes (obtained by LPE) onto HOPG substrates (herein referred to as BF BP-C(HOPG) electrodes, see methods and Figure S2 in the Supporting Information for further sample prep information). HOPG was chosen as the conductive carbon composite in our EC-AFM experiments due to its low surface roughness. This surface provides a smooth and well-defined background that helps in obtaining high-resolution and artifact-free images of the BF BP-C(HOPG) electrode during EC-AFM. Additionally, the adherence of BP particles to the surface of HOPG following the introduction of electrolyte solution is indicative of robust in-plane vdW interactions arising from the induced electromagnetic interactions between the two two-dimensional (2D) materials.^56^ A schematic of the EC-AFM electrochemical cell is shown in Figure S3. Optical microscope (OM) images of the final BF BP-C(HOPG) electrodes at OCV captured during EC-AFM are shown in Figure S4 (Supporting Information), showing a range of BP particle sizes (surface area of flakes nominally <10 × 10 μm^2^). This is to be expected as mechanical exfoliation of BP commonly yields nanoflakes of various heights (0.7–200 nm)^57^ and lengths (typically ∼1–10 μm), since the process is not entirely controlled, and the number of layers peeled off can be stochastic.

EC-AFM topography images taken of a single BP flake on HOPG while under electrochemical control vs Li/Li^+^ in the range 3.0–0.01 V are shown in Figure 3. The full series is reported in Figure S7 with images at OCV (Figure 3a) and selected voltages (Figure 3b–d) shown in Figure 3. Here, the black arrows indicate the direction of the AFM scan, noting that each image dynamically captures 1 V. To study the structural evolution and SEI growth more precisely, the height across a selected HOPG edge, HOPG basal plane, and BP basal plane was measured during the first cycle, taken from points 1–3 in Figure 3a respectively, and are plotted in Figure 3e,f. To do this, the feature heights were measured relative to the lower basal plane for both BP and HOPG (Figure S5). The CV from the BF BP-C(HOPG) EC-AFM electrode cell is presented in Figure S6 (Supporting Information), where it is compared to an equivalent cell with pure HOPG, and coin cells containing binder inclusive BP-C(graphite). The acquired CVs exhibit the characteristic BP features at similar potentials to those observed in the coin cells (Figure 1), although the background contribution from HOPG was more significant due to the lowered BP loading in BF BP-C(HOPG).

We first consider the HOPG surface and accompanying SEI growth during EC-AFM. At OCV (Figure 3a), the HOPG step edge height was measured to be 8.7 nm in height corresponding to 24 or 25 layers of graphene (∼0.335 nm per layer).^58^ Between OCV and 2 V (Figure S7a,b), no significant topographical changes were observed, but as the potential was swept from 2–1 V a small increase in the HOPG step edge height was seen (Figure S7c). This arises due to the initial formation of SEI, and is consistent with the electrochemical response measured between 1.75 and 1.25 V for HOPG reported in Figure S6b and literature.^59,60^ We note that it is well understood that lithium ions only intercalate into graphite layers through the edge plane rather than the basal plane.^60,61^ Consequently, this increased flux drives more interphasial species to accumulate at step edges, resulting in an inhomogeneous SEI across the HOPG surface. This becomes more apparent between 1.0–0.01 V (Figures 3b and S7d) where a large accumulation of SEI species at the HOPG edge can be seen. The SEI layer grown at the HOPG basal planes was found to be much thinner than that on the step edges, which is consistent with previous reports of preferential SEI formation at the step edges of graphite.^60,62^ The height of the HOPG step edge (point 2), tracked in Figure 3e (blue) increases from 10 nm (Figure S7c) to 221 nm (Figure S7d). Whereas in comparison, SEI growth at the HOPG basal plane (point 1, tracked in Figure 3e (red)) is limited, increasing from 12.4 nm (Figure S7c) to 14 nm (Figure S7d). In addition, particle-like structures simultaneously form on the HOPG basal plane, producing a rougher surface. The accumulation of SEI at step edges and a roughening of the basal plane from SEI particle formation on HOPG is likely to reduce the adhesive forces between BP and HOPG, promoting BP detachment.

Next, we consider the BP topography and accompanying SEI growth. Particular attention is paid to the BP flake height during EC-AFM. BP lithiates via an intercalation-alloying mechanism, with intercalation (which preferentially occurs via the layer channels) and the alloying aspect both resulting in a significant volume expansion of ∼300% for the Li_3_P compound.^63^ Further, any changes to the BP-C vdW adhesion interaction during lithiation will be captured in this parameter. To investigate this lithiation-induced expansion, the height of the BP flake was measured and plotted individually in Figure 3f. Initially, at OCV (Figure 3a) the BP flake height was measured to be 9.2 nm thick, corresponding to ∼17 phosphorene layers (∼0.525 nm per layer).^64^ Significant changes in BP flake height were observed during discharge in the 1.0–0.01 V range (Figure 3b), most drastically between 0.6–0.4 V, where the BP flake increased in height to 120 nm. This corresponds to a large height increase of ∼1250% from OCV. In part, this expansion is attributed to the growth of SEI species on the Li_*x*_P_*y*_ surface and Li_*x*_P_*y*_ volume expansion due to the initial alloying of phosphorus to lithiation stoichiometries up to Li_2_P. But, given that the magnitude of expansion (∼1250%) is significantly larger than reported for high-lithiation states of Li_*x*_P_*y*_ (∼300% for Li_3_P^20^), this expansion also contains contribution from the BP flake lifting/curling during lithiation, and partially beginning to detach from the HOPG surface. Indeed, as the potential is swept <0.4 V (Figure 3b) the flake completely detached (Figure 3c), leaving behind the HOPG surface (Figure 3d). This is not an isolated incident; both Figures S8 and 4 show other examples of BP detachment.

**Figure 4:**
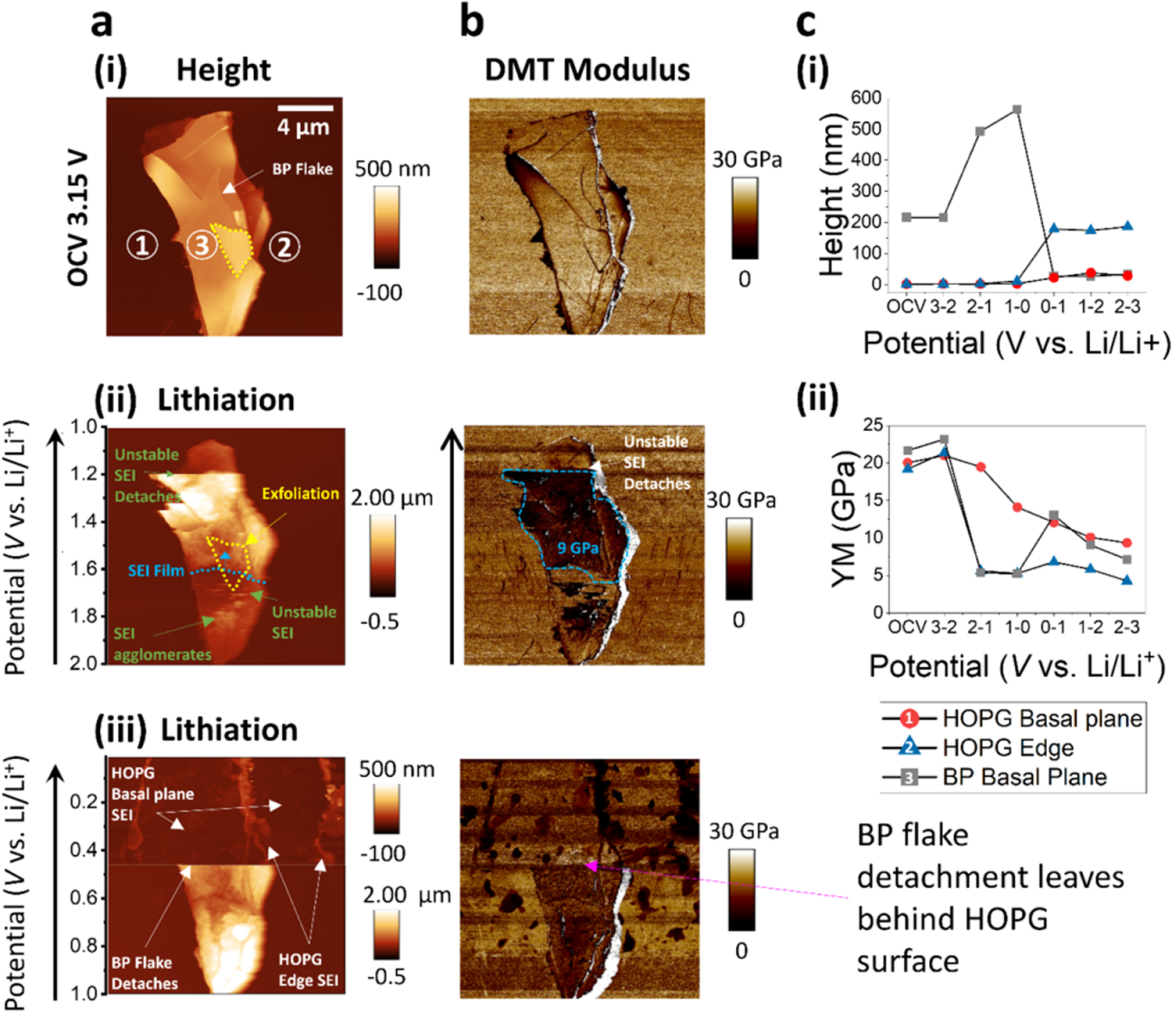
EC-AFM imaging with DMT modulus maps of a binder free BP-C(HOPG) anode collected while under electrochemical control in the range 3–0.01 V at 0.5 mV s^–1^ in 1 M LiPF_6_ EC/DEC electrolyte vs Li/Li^+^. Selected EC-AFM (a) height images and (b) DMT modulus maps: (i) OCV, (ii) 2.0–1.0 V during discharge (lithiation), (iii) 1.0–0.01 V during discharge (lithiation). The full series is reported in Figure S9, Supporting Information. All EC-AFM images captured across 20 × 20 μm^2^ area, voltages plotted vs Li/Li^+^, with the *z*/modulus scalebar reported to the right of the respective image, and *x*–*y* scalebar inset in (a(i)). The black arrow corresponds to the AFM scan direction. Clear SEI exfoliation and detachment of the SEI can be seen in (a(ii)). The BP flake detaches in (a(iii)), leaving behind the HOPG surface. (c) The tracked relative (c(i)) heights and DMT modulus (c(ii)) of a HOPG basal plane, HOPG step edge, and BP basal plane (labeled 1, 2, and 3 respectively in (a(i))) as a function of the AFM image potential window.

The large expansion of BP driven by lithiation introduces significant interfacial stress between BP flakes and HOPG. This strain weakens the bonding between BP and HOPG, ultimately causing detachment or delamination of the BP layers from the substrate. We note a similar phenomenon is reported for pure HOPG, where layers of graphite have been shown to delaminate from the increased interlayer distance induced by the intercalation of solvated ions during lithiation.^65^ It was also previously discussed that SEI accumulates at the step edges of HOPG, further reducing the adhesive forces between BP and HOPG, which falls within the potential window of the alloying mechanism of Li into BP that also induces a large volume expansion. Therefore, the combination of these mechanisms presents a compatibility issue for BP-C composite electrodes. This incompatibility ultimately drove the detachment of BP from HOPG.

As far as we are aware, the combined impacts of the incompatibility of SEI growth on carbon, in conjunction with the destabilizing of BP during intercalation-alloying transitions, is a degradation mechanism for BP-C composite electrodes that has not been reported to date, and one that must be overcome if BP anodes are to be stabilized. When considering the application of these composite anodes in LIBs, structural instability and incompatibility across the BP-C interface could present additional challenges. It has been established that the BP-C binding alone is not sufficiently able to withstand BP expansion, resulting in the dissociation and isolation of deactivated BP, which significantly decrease the overall performance and cycle life of the composite. This detachment not only hampers the electrical conductivity between the composite active material in LIBs, but also disrupts the intimate contact necessary for efficient electron and ion transport, resulting in a rapid capacity fade and diminished energy storage capabilities of the battery system. Indeed, this is consistent with Figures 1 and 2, which showed the capacity dropping and resistance increasing for binder inclusive BP-C composite electrodes during multiple charge–discharge cycles. In addition, these results highlight that a mismatch in the SEI morphology across the BP-C interface is also an important factor in the worsening long-term electrochemical performance of these composites. The accumulation of SEI between the BP flakes and carbon surface interface has also shown to drive BP isolation. In the context of binder inclusive BP-C composites applied in LIBs, this could also affect the charge transfer rate between the BP and C, and between the composite and electrolyte. This could lead to increased charge transfer resistances, hence limiting the rate capability of the material. Therefore, addressing this challenge is crucial for the development of stable and high-performance composite anodes.

### Investigating the Evolution of the Mechanical Properties at the Electrode/Electrolyte Interface of Binder Free BP-C(HOPG) Electrodes

In the EC-AFM discussion above (Figure 3), it became evident that the growth of the SEI on HOPG and BP, combined with BP volume expansion during cycling, resulted in the detachment of active material. Therefore, it is crucial to comprehend the mechanical properties at the electrode/electrolyte interface, particularly in drawing comparisons between BP and C, to gain insights into these phenomena. Consequently, alongside EC-AFM analysis of the morphological evolution of BF BP-C(HOPG) composites, the mechanical properties were investigated to deepen our understanding of electrochemical mechanisms and degradation processes (Figures 4 and S9, Supporting Information).

Figure S9 (Supporting Information) presents a series of EC-AFM images of the BF BP-C(HOPG) composite when discharged from 3.0 to 0.0 V and then charged to 3.0 V. Selected images from this series are shown in Figure 4. The height maps (Figure 4a), and Derjaguin–Muller–Toporov (DMT) modulus (Young’s modulus calculated according to the DMT model, explained in the Supporting Information) maps (Figure 4b) are presented within a square scan area of 20 × 20 μm^2^, where a single BP flake is observed on the HOPG surface. The black arrows indicate the direction of the AFM scan, where each image captures 1 V. The topography of the HOPG step edges can be more clearly seen in Figure S9b where the *z*-scale is adjusted to capture smaller variations in height of the HOPG. To establish the relationship between morphology and mechanical properties, measurements were taken at specific points on the HOPG step edge, HOPG basal plane, and BP basal plane (from the points 1, 2, and 3 respectively identified in Figure 4a(i)), and are plotted in Figure 4c(i,ii). Given that the voltage is not constant across the line scan, the height and DMT modulus are plotted as a function of the voltage range in which the measurements were taken. The feature height was measured relative to the normalized lower HOPG basal plane, as shown in Figure S10 at OCV. The measurements were replicated across each EC-AFM image in the series as the potential was swept (Figure S9, Supporting Information).

**Figure 5:**
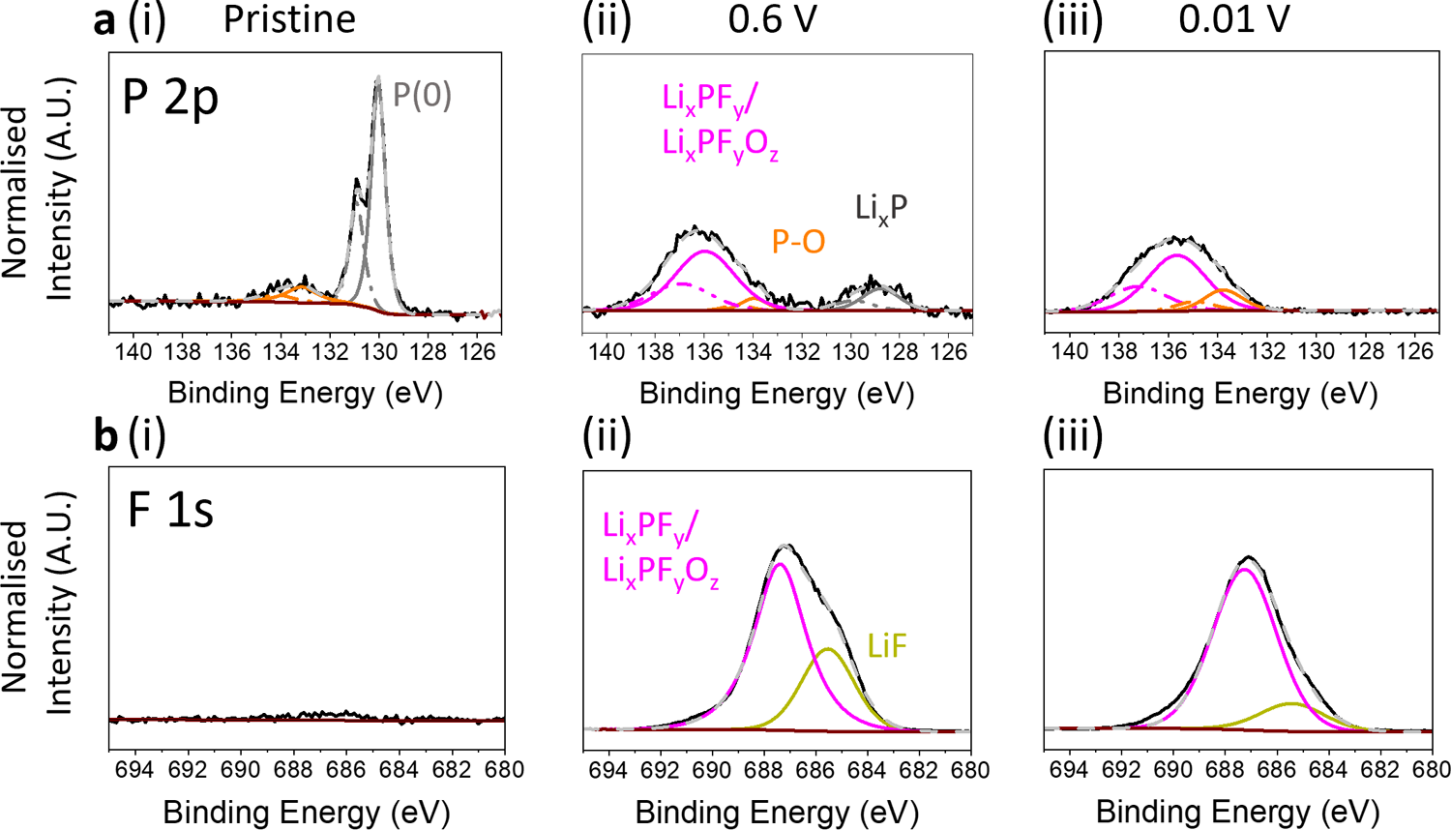
High resolution XPS spectra of binder free BP-C(HOPG) electrodes. (a) XPS P 2p spectra and (b) F 1s spectra of (i) pristine binder free BP-C(HOPG), (ii) BF BP-C(HOPG) anode discharged and charged between 2.5–0.6 V, and (iii) 2.5–0.01 V vs Li/Li^+^. All XPS spectra are normalized with respect to the P(0) peak in (a(i)).

The first row of images in Figure 4 displays the morphology and DMT modulus maps of the BF BP-C(HOPG) composite surface at OCV, which contains a single flake of BP deposited onto the freshly cleaved HOPG surface. Here, the height of the HOPG step edge (point 1) is 1.46 nm. The DMT moduli at the HOPG step edge (point 1) and HOPG basal plane (point 2) were measured as 19.2 and 20.0 GPa respectively, close to the theoretical value of graphite (18 GPa).^66^ The height of the BP flake shown in Figure 4a(i) was found to be 216 nm (corresponding to ∼415 layers). The DMT modulus across the BP basal plane was measured at an average of ∼22 GPa. Previous reports by AFM-nanoindentation measurements determined the average value for the three-dimensional (3D) Young’s Modulus of BP to be 41 ± 15 GPa (under vacuum), without accounting for the flake thickness, and crystallographic orientation.^67^ While our measured value is slightly lower than that reported in literature for freshly cleaved bulk BP under vacuum, it is worth noting that the Young’s modulus of BP layers are sensitive to the measurement environment, as this value can depreciate significantly with exposure to ambient temperatures, and is sensitive to the presence of surface defects.^68^ Therefore, we attribute the differing moduli to low level surface oxidation (which is confirmed with X-ray photoelectron spectroscopy (XPS), Figure 5), arising from the high reactivity to trace oxygen in the glovebox atmosphere, potential oxidation/spontaneous SEI growth upon contact with the liquid electrolyte, as well as the presence of surface defects, and surface terraces. A lower DMT modulus was measured for the terrace edge and defect site (taken from points shown in Figure S11) and found to be ∼3 and 5 GPa, respectively. Nevertheless, at OCV, the mechanical properties of BP and C fall within the same range, and therefore are well matched.

During the cathodic scan, significant changes in the morphology and DMT moduli of the BP and C surface were seen. For BP, at ∼1.9 V the growth of a small number of discrete and soft nanoparticles were observed to grow around the defective regions (Figure 4a(ii), enlarged and highlighted in green-dotted circles in Figure S11c, Supporting Information), indicating a localized stress response to the defects. The average DMT modulus of the nanoparticles around the structural defect at ∼1.9 V were recorded at ∼7 GPa, (Figure S11, Supporting Information). This assessment is supported by the increased density of these SEI particles, forming large, unstable and soft accumulation of SEI species at ∼1.8 V (highlighted in Figures 4a(ii), S11c and S12), that had an average DMT modulus of ∼2 GPa. These soft SEI deposits were unstable, which resulted in partial SEI detachment from the BP surface. Nevertheless below 1.6 V, SEI particles continued to accumulate and formed a distinct softer layer (∼9 GPa) (shown by the blue dotted line in Figure 4a,b(ii)), and at ∼1.4 V the height increased to ∼490 nm (from ∼200 nm initially at OCV), corresponding to 145% expansion. This resulting layer was also unstable, where it could be seen to become partially removed from the BP surface at ∼1.2 V. The correlation between SEI detachment and softer SEI regions is significant as it suggests that regions with weaker interfacial bonding are more prone to detachment. Similarly, within the same potential range it observed that upper surface layer(s) of BP exfoliated (highlighted by the yellow dotted line in Figure 4a(i,ii)), leaving the underlying SEI covered BP plane. At OCV, this surface terrace had a lower DMT modulus at the BP terrace edge of ∼3 GPa, compared to 22 GPa at the BP basal plane. The interphase detachment at softer regions at the edge of the exfoliated surface terrace further confirms that regions with weaker interfacial bonding are more prone to detachment.

BP-lithiation during intercalation-alloying also drives significant structural changes of the BP flake, which alter the mechanical properties of the material. This can be seen in Figure S12, where the accumulation of an SEI film below 1.6 V, was accompanied by the propagation of long, thin and unidirectional wrinkles. This phenomenon arises from lithiation-induced compressive stresses, compensated for by a stretching of the longer P–P bonding, which manifest as linear/anisotropic distortions of the BP sheet.^32,64^ The height and width, versus the linear DMT across these wrinkles were also reported in Figure S12. The wrinkles appeared to increase in height with decreasing potential, i.e., their volume expansion increased with increased lithium concentration. The average DMT modulus across the length of the wrinkles was measured at ∼15 GPa. This was lower than the reported OCV DMT modulus (22 GPa), corresponding to a 38% reduction, which could indicate a combination of the presence of a soft SEI layer and/or the formation of a softer intermediate Li_*x*_P_*y*_. Although there have been no experimental reports that directly evidence this, theoretical studies have shown that lithiation of BP leads to a softening, reducing the modulus by 56% from bulk to Li_2_P,^69^ which is in line with these results. Ultimately, this demonstrates that there is softening at the BP-C-electrolyte interface during formation of the SEI and Li_*x*_P. Comparatively, within this operational window, (2.0–1.0 V), the underlying HOPG modulus begins to decrease, where SEI species grow. The HOPG modulus decreased to 6 GPa at the edge and 17 GPa at the basal plane. This is crucial as dynamic variations in the DMT modulus at the (BP-C)-SEI interface imply different levels of stiffness across the SEI layer formed between Li_*x*_P_*y*_ and C. This mechanical mismatch can lead to stress concentrations, which over time can induce cracking, delamination, or other unwanted structural defects in the composite, compromising its mechanical integrity.

Indeed, further lithiation between 1 and ∼0.5 V results in the continual anisotropic volume expansion of the BP flake, as well as SEI reformation, resulting in an interface with a low DMT modulus of ∼10 GPa at 0.8 V (Figure 4b(iii)). After which point, at approximately ∼0.46 V, the flake detaches from the surface (labeled in Figure 4a(iii),b(iii)). During the same operational window, 1–0.01 V, HOPG edge and basal plane DMT moduli decreased further, with more significant decrease at step edges (13 and 5 GPa for basal plane and edge respectively (Figure 4c(i))). This mechanical behavior contrasts notably with lithiated BP, which exhibits less variation across its entire plane.

This discrepancy in mechanical behavior becomes crucial for understanding the interfacial delamination of BP, where the mechanical properties of the SEI layer formed between BP-C play a pivotal role, influencing the adhesion and ultimately leading to the reported interfacial delamination phenomenon. A parallel behavior has been previously documented in HOPG, where intercalation-induced degradation of soft SEI products between layers can induce partial or complete exfoliation of overlaying layers.^65^ Indeed, after BP detachment and in the reverse scan (Figure S9a(iv–vi), Supporting Information), the underlying HOPG surface exhibits similar SEI morphology and mechanical properties to that grown in the absence of BP.

These EC-AFM observations strengthen the hypothesis that variations in SEI quality and compositional heterogeneities across the BP-C interface directly influence the mechanical stability of the composite material. We show that the dynamic changes in DMT modulus cause variations in the strain experienced by the composite materials during charge and discharge cycles. Since BP and HOPG exhibited significantly different DMT modulus values at any given point during cycling, as well as variation in the homogeneity across the interface of each, this generates interfacial strain at the composite interface, which ultimately contributed to the detachment of BP particles from the HOPG substrate (as seen in Figure 4a(iii)). Crucially, linking these phenomena to the electrochemical performance as determined in Figures 1 and 2, provides deeper understanding of the poor capacity and cycling stability experienced by these composites when applied to electrochemical devices. For example, unstable and mechanically mismatched interfaces therefore lead to increased charge transfer resistance, electrolyte penetration, and active material isolation during cycling, as well as continual electrolyte decomposition. Therefore, knowledge of electrode/electrolyte interfacial properties could guide the selection of composite materials with compatible properties, or strategies, to ensure a strong and uniform interface between BP-C, preventing delamination or detachment during the battery’s operational life.

### Characterizing the Chemical and Structural Composition of the SEI/BP-C Interface

To elucidate the correlation between the Li^+^ ion storage mechanisms and the interphasial properties for BF BP-C(HOPG) composite electrodes, the chemistry of the SEI was investigated using XPS (with inert transfer). First, to determine the binding interaction between BP and C in the pristine electrodes, Figure S13 compares the C 1s and P 2p spectra of the as prepared BF BP-C(HOPG) electrodes to pristine HOPG. These spectra indicate the XPS peaks of the P 2p and C 1s edges were not significantly affected by the deposition process.^70^ Thus, this confirms that no chemical reaction occurred during the synthesis and that the heterostructure was held together by vdW forces, as has been reported previously for BP-C electrodes formed via self-assembly.^32^

To determine the influence of BP-alloying on the final BP-C SEI composition, the BF BP-C(HOPG) electrodes were cycled within a controlled operational window. Figures 5, S14 and S15 (Supporting Information) highlight the differences in SEI chemical composition for BP electrodes in the discharged state, after they had been cycled pre-BP alloying, between 2.5–0.6 V, and post-alloying between 2.5 and 0.01 V, and show a comparison to the pristine BF BP-C(HOPG) electrode. The electrodes were carefully removed from the EC-AFM cell in an air free glovebox environment, rinsed in DEC and finally dried under vacuum overnight prior to analysis.

The P 2p spectra are compared in Figure 5a(i–iii). In the P 2p spectrum for the pristine BF BP-C(HOPG) (Figure 5a(i)), the P(0) 2p region can be observed as a pair of spin–orbit split components at 130.04 eV (P 2p_3/2_) and 130.94 eV (P 2p_1/2_), alongside a small contribution at higher binding energies (P 2p_3/2_ = 133.04 eV, P 2p_1/2_ = 134.12 eV) attributed to phosphate groups (P–O), indicating low-level surface oxidation of BP, which is unsurprising due to its significant oxygen sensitivity.^71^ After electrochemical cycling to 0.6 V (Figure 5a(ii)) a new pair of peaks centered at ∼137.2 eV were seen to develop from the presence of fluorinated phosphorus species, such as Li_*x*_PF_*y*_O_*z*_ and/or Li_*x*_PF_y,_ suggesting the formation of a surface layer originating from the reductive decomposition of PF_6_^–^ anion.^72^ The remaining presence of elemental P(0) peaks after cycling the electrode to 0.6 V cutoff voltage, implies that BP is not fully passivated with an SEI layer, which is consistent with the formation an partially formed/removed SEI, such as the unstable SEI growth shown in Figure 3. Additionally, the peaks were shifted to a lower energy 128.81 (P 2p_3/2_) and 129.87 eV (P 2p_1/2_), which have binding energies ∼1.4 eV lower than those fitted for the BP starting material and are therefore likely to also encompass a contribution from the reduction product of P, Li_*x*_P.^14,27^ The downshifted binding energy is attributed to electron donation to the phosphorus host crystal, which reduces the binding energy of valence P 2p electrons. The presence of this binary compound, above alloying potentials, suggests that electrically isolated and reduced material was generated from the morphological reordering of BP-C interface during the initial stages of lithiation of BP, as well as the growth of incompatible SEI layer between the BP-C junction. Importantly, after the BP was cycled below alloying potentials, 0.01 V, (Figure 5a(iii)), the P(0) peak disappeared, suggesting removal of BP from the HOPG surface, which is in good agreement with the EC-AFM data, that shows the BP-C covalent bonding/vdW interactions alone are not enough to prevent electrode degradation.

Figure S14 (Supporting Information) shows the C 1s, O 1s and Li 1s spectra for the composite BF BP-C(HOPG) electrodes in the pristine form (Figure S14a–c(i) (Supporting Information)), cycled to 0.6 V, and 0.01 V (Figure S14b (Supporting Information)) respectively. The primary components of the SEI layer were determined to be loosely packed organic-rich species such as ROCO_2_Li, (CH_2_OCO_2_Li)_2_ and polycarbonates, and inorganic species including Li_*x*_PF_*y*_ and/or Li_*x*_PF_*y*_O_*z*_, LiF, Li_2_O and Li_2_CO_3_.The results are consistent with similar reduction products Li electrolytes.^54,73−76^ Related comparisons of the SEI for LIBs have been recently reported and are presented in Table S1 (Supporting Information).

The F 1s spectral peaks for the electrodes cycled to 0.6 and 0.01 V, shown in Figure 5b(ii,iii), further confirm that the SEI was partly comprised of inorganic components. The peak at ∼687.2 eV was likely to derive from fluorinated phosphorus species, Li_*x*_PF_*y*_ and/or Li_*x*_PF_*y*_O_*z*_, and those at ∼685.3 eV from LiF. Interestingly, the relative signal intensity of the LiF species increased with deeper electrochemical cycling for the BP-C composite. Since it is generally accepted in literature that SEI is composed primarily of an inner layer of inorganic compounds and an outer layer of organic species,^74^ the increase in intensity of the LiF could arise from the displacement of underlying inorganic SEI layers from the expansion and detachment of BP during alloying, which is consistent with previous BP-based reports.^14^ For comparison, the F 1s spectra for the bare HOPG electrode cycled down to 0.01 V (Figure S15d(ii)) showed similar relative intensities of the LiF peak to the BP-C(HOPG) electrode cycled above alloying potentials, 0.6 V, prior to BP detachment. These results, in addition to the EC-AFM presented, shed light on the inability of the SEI layer and BP-C hybridization to withstand the significant structural changes that are accompanied by the disintegration of active BP material during alloying.

## Conclusions

Here EC-AFM imaging has revealed significant incompatibilities between the morphology of the SEI growth on carbon and the destabilization of BP during lithiation processes. Furthermore, investigations into the mechanical properties of the BP-C interface highlighted dynamic variations in stiffness across the SEI layer, contributing to BP-C interfacial strain, cracking, and delamination of BP during charge and discharge cycles. These results were corroborated with XPS, which identified the presence of electrically isolated active material resulting from the irreversible lithiation of BP. Finally, this degradation mechanism was also shown to lead to compromised electrical conductivity, inefficient electron/ion transport, and a rapid decline in capacity and energy storage capability of BP-C composites. Hence, the simultaneous nature of the SEI growth on carbon and BP structural changes represents a critical challenge to composite stability, driving BP and carbon to detach from one another. Together the data presented offer valuable insights into the factors influencing the poor long-term performance of BP-C composites, highlighting the pivotal role polymeric binders play in the stabilization of BP-C composite anodes. The identified degradation mechanisms emphasize the need for tailored strategies to address interfacial stability, compatibility issues, and mechanical integrity in composite electrode materials. As we strive toward efficient energy storage systems, further research is warranted to unlock the full potential of BP-based anodes, and addressing these challenges is crucial for the realization of stable and high-performance LIBs using BP-based anodes.

## Methods

### CV Tests of Electrochemical Cells and Coin Cells

Three types of electrodes were prepared for this study: binder inclusive BP-C(C45), binder inclusive BP-C(graphite), and binder free BP-C(HOPG). Macroscopic crystals of BP (99.998% purity) from SmartElements were used to make all electrodes. The as received BP was crushed by mixing with a mortar and pestle for 30 min, transferred to a glass-metal transition tube (part number KSEG-150, MDC Precision) and attached to a Pfieffer turbopump to be outgassed to pressures less than 1 × 10^–6^ mbar while being heated to 100 °C in a tube furnace.

For the CV testing CR-2032 coin cells were constructed inside an argon filled glovebox with binder inclusive BP-C electrodes (9 mm diameter discs), electrolyte (1 M LiPF_6_/EC/DEC (1:1 (v/v))) and Li metal (16 mm diameter chip, Alfa Aesar 99.95% (metals basis)), separated by a polyethylene separator (2320, 20 μm thickness, Celgard, 19 mm diameter). The BP-C(graphite) composite was formed with a 60/40 mass ratio mix of the phosphorus sample with graphite (EQ-Lib-CMSG, MTI Corporation) that was mixed using a mortar and pestle for 30 min. To fabricate the binder inclusive BP-C(graphite) electrodes, the BP-C(graphite) was coated onto copper foil (9 μm thickness, MTI Corporation) via a slurry in 1-methyl-2-pyrrolidinone (NMP, Merck, anhydrous, 99.5% purity) with 10% solid mass of poly(vinylidene fluoride) binder (PVDF—Solef 5130), such that the overall mass ratio of P/C/PVDF was 54:36:10. The fabricated electrodes were dried under dynamic vacuum at 120 °C for 24 h prior to coin cell construction. The resulting coat-weight of the binder inclusive BP-C(graphite) electrodes was 9.47 g m^–2^ with a corresponding coating thickness of 100 μm. The electrolyte used was 1 M LiPF_6_ salt dissolved into EC/DMC (3:7 vol) solvent mix (Soulbrain). CV measurements were made using a Gamry Interface 1000 potentiostat, using a scan rate of 0.2 mV s^–1^ with a 0.2 mV step size; scanning from 2 to 0.001 to 2 V.

For the CR-2032 coin cells testing, BP-C(C45) electrodes were constructed from exfoliated BP (45%) Super C45 (45%) and poly(vinylidene fluoride) (PVDF—Solef 5130) (10%) on copper foil. Coin cells were assembled versus a lithium metal counter electrode (Alfa Aesar 99.95% (metals basis)) and contained electrolyte (1 M LiPF_6_/EC/DEC (1:1 (v/v))) and a polypropylene separator (Celgard, 9 mm diameter). For these coin cells, BP was outgassed for 1 week, then exfoliated via a liquid-phase exfoliation (LPE) method adapted from a procedure reported previously.^32^ BP was dispersed in *N*,*N*-dimethylformamide, anhydrous (NMP—99.8% (Merck)) (cylindrical vial, 20 mL NMP) at a concentration of 0.1 mg mL^–1^ in an argon filled glovebox. The vials were sealed, removed from the glovebox and sonicated in an ultrasonic bath (Ultrawave QS3, 50 W) for 12 h with the bath water changed every 20 min in order to keep the water temperature below 40 °C. The resultant dispersion was transferred back to the glovebox and into a sealed Buchi vessel (B-585 Drying). The solution containing Buchi was left under vacuum and heated at 80 °C for 1 week to evaporate the majority of the NMP. The residual filtrate was then scraped into a cylindrical vial and placed in a glass-metal transition tube where it was evacuated further to <10^–6^ mbar using a turbomolecular pump and left under dynamic vacuum (continuous pumping) for 1 week, before the temperature was increased to 100 °C for a further week, leaving behind a powder of exfoliated BP. A typical slurry was made from carbon black (EQ-Lib-Super C45) and the BP powder and PVDF binder in a mass ratio of 45:45:10, mixed in NMP manually via pestle and mortar. The mass loading of active material (BP) was ∼0.460 mg cm^–2^, corresponding to a total mass loading of ∼1.01 mg cm^–2^ and a thickness of ∼6.5 μm.

Electrochemical data (charge–discharge and EIS) were collected using a BioLogic BSC-805 battery cycler within the potential range 0.01–2.5 V(versus Li/Li^+^) at 0.2C (0.05 A g^–1^), with the BP-C/Li CR-2032 coin cells as assembled as above. The specific capacity was calculated based on the weight of phosphorus. For EIS tests, the coin cells were discharged–charged between OCV and 0.01 V for 1 cycle, with a constant current density of 0.2C (0.05 A g^–1^). EIS were taken at open circuit potential, 2.0, 1.6, 1.5, 1.4, to 1.0, and 0.01 V. The potentiostatic EIS test was set from frequency of 100 kHz to 0.01 Hz, at AC voltage of 10 mV. The Nyquist plot of the EIS were obtained and fitted with RelaxIs- rhd instruments software.

For the AFM cell, BP was mechanically exfoliated via LFP (as described above), after which 20 μL of the dispersed solution was dropcast onto the HOPG substrate to form binder free BP-C(HOPG) electrodes. These were then and placed in a glass-metal transition tube where they were evacuated to <10^–6^ mbar using a turbomolecular pump and left under dynamic vacuum (continuous pumping) for 1 week, before the temperature was increased to 100 °C for a further week. A schematic showing electrode fabrication can be seen in the schematic in Figure S2 (Supporting Information). The substrates used were HOPG (ZYM grade, 12 × 12 mm^2^, 1 mm thickness, Bruker Corp.) The electrode area was defined using an adhesive polyimide film (Kapton) punched with a 5 mm diameter hole. The counter and reference electrodes were two separate Ni wires wrapped with lithium foil, which were placed near the working electrode inside the electrolyte (1 M LiPF_6_/EC/DEC (1:1 (v/v))). A schematic of the EC-AFM can be seen in Figure S3 (Supporting Information).

### Structural Characterization

EC-AFM (Bruker Dimension Icon with ScanAsyst) experiments were carried out in an Ar-filled glovebox (Mbraun YKG series) with H_2_O < 0.1 ppm, O_2_ < 0.1 ppm combined with a CH Instruments electrochemical workstation (Model 700E Series Bipotentiostat). The film morphology was characterized using PeakForce Qantitative Nanomechanics (QNM) tapping mode with a RTESPA-525 silicon probe with a reflective Al coating (Bruker Corp., *k* = 200 N m^–1^, f_0_ = 525 kHz). All the results obtained from the AFM were analyzed by Gwydion software. PeakForce QNM tapping mode was utilized to image the electrodes in fluid. In this mode the cantilever oscillates, far below the resonant frequency, and the vertical motion of the cantilever using the main piezo element (Z) relies on the feedback force. The real feedback loop maintains a constant maximum interaction force (peak force) between the probe and the sample surface at each pixel, to obtain topography of that sample. This method can provide atomic level resolution at low imaging forces, preserving the sample and tip, enabling imaging of the delicate soft SEI layers with high accuracy. This mode also reduces interference during liquid phase imaging, compared to tapping mode as it does not require the probe to oscillate at resonance frequency.^36^ The DMT modulus was calculated using the same QNM PeakForce tapping mode with the RTESPA-525 silicon probes with reflective Al coating (Bruker Corp., *k* = 200 N m^–1^, f_0_ = 525 kHz) and using the relative method to calibrate against HOPG (18 GPa).

### XPS Characterization

After EC-AFM electrochemical tests, the BP electrodes were taken from the EC-AFM cell and rinsed with DEC to remove any residual electrolyte salt, followed by drying for 24 h in the glovebox at ambient temperature. Surface analysis was carried out with X-ray photoelectron spectroscopy (Thermo Scientific K_α_). The spectra were collected at room temperature using monochromaric Al–K_α_ (1486.6 eV) radiation as an incident X-ray source. The electrodes were placed on a sample holder with carbon conductive tape in an argon-filled glovebox. The sample holder was introduced into a load-lock chamber using a transfer vessel (Thermo Scientific 831–57–100–2) without air exposure. A background correction and normalization (relative to the strongest XPS photopeak in the series) was applied to all XPS spectra.

## Supporting Information Available

EIS spectra and circuit model fitted values; schematic of BP-C(HOPG) formation; schematic of EC-AFM cell; optical microscopy images of BP-C(HOPG) electrodes; additional EC-AFM images including full series of Figures 3 and 4, plus analyzed images; CVs BP-C(HOPG) and HOPG taken with EC-AFM cell; additional XPS data (PDF)
