# Supplementary material for: Electrochemical Atomic Force Microscopy of Black Phosphorus Composite Anodes: Electrode Destabilization and Degradation Mechanisms in Alkali-Ion Batteries
Source: ACS Appl Mater Interfaces. 2024 Aug 7;16(33):43512–25. doi: 10.1021/acsami.4c06693 (PMC11345720; doi:10.1021/acsami.4c06693)
Supplement: Supplementary file 1 — am4c06693_si_001.pdf [file am4c06693_si_001.pdf]

Supporting Information

Electrochemical Atomic Force Microscopy of Black Phosphorous Composite Anodes: Electrode Destabilisation and Degradation Mechanisms in Alkali-Ion Batteries

Samia Said[1], Rebecca R. C. Shutt[2], Zhenyu Zhang[1,3,4], Adam J. Lovett[1,3] , Christopher A. Howard[2], Thomas S. Miller[1,3*].

[1]Electrochemical Innovation Lab, Department of Chemical Engineering, University College London, Torrington Place, London, UK, WC1E 7JE.

[2]Department of Physics & Astronomy, University College London, Gower Street, London, UK, WC1E 6BT.

[3]The Faraday Institution, Quad One, Becquerel Avenue, Harwell Campus, Didcot, Oxfordshire UK, OX11 ORA.

[4] Renewable Energy Group, Department of Engineering, Faculty of Environment, Science and Economy, University of Exeter, Penryn Campus, Cornwall, UK, TR10 9FE.

*Corresponding author: t.miller@ucl.ac.uk

Electrical Impedance Spectroscopy Studies of Binder Inclusive BP-C(C45) Composites

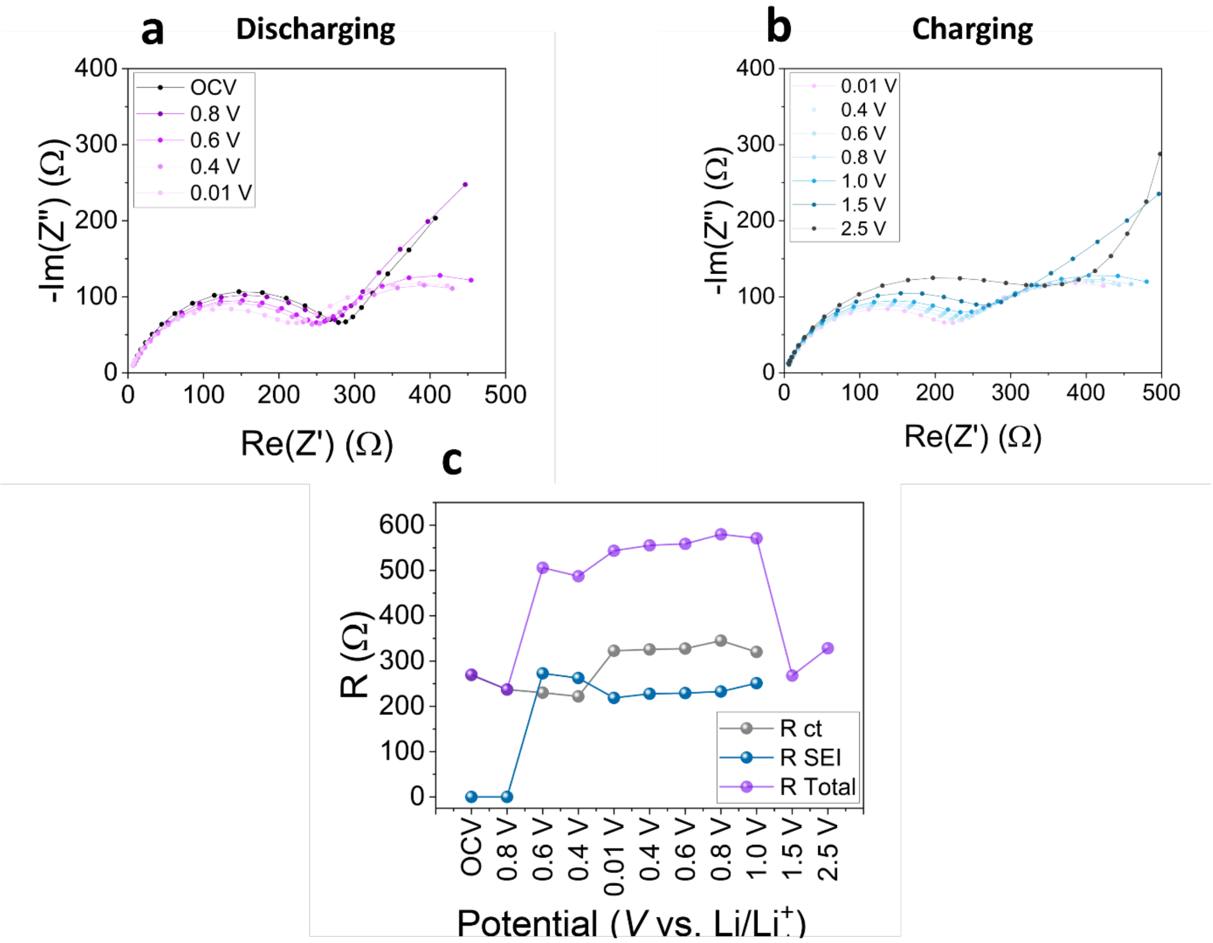

Figure S1: Electrical impedance spectroscopy study of binder inclusive BP-C(C45) electrodes collected at different cell potentials during the 1st (a) dicharge and (b) charge. (c) Fitted resistance values of the SEI film, charge transfer processes, and sum of total resistances ($R_{Total} = R_{Bulk} + R_{ct} + R_{SEI}$).

Preparation of Binder Free BP-C(HOPG) electrodes by Liquid Phase Exfoliated BP.

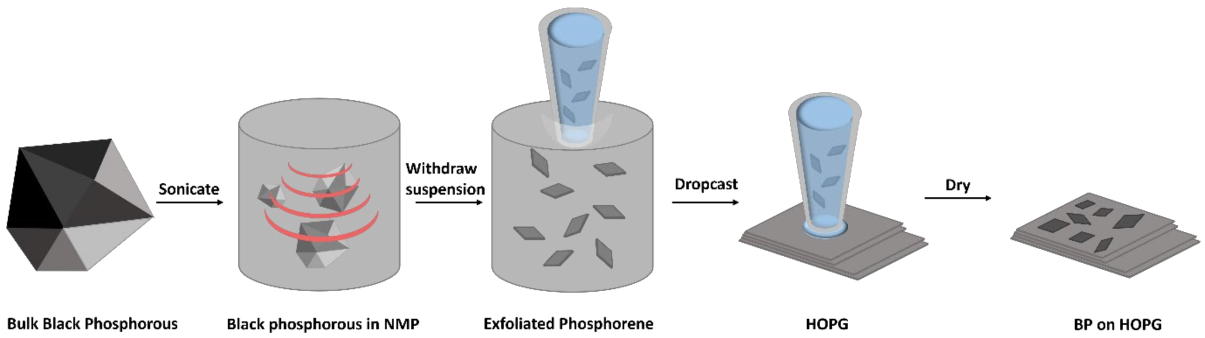

Figure S2: Schematic diagram showing the formation of liquid phase exfoliated BP (LPE BP), followed by deposition and drying onto HOPG to form the binder free BP-C(HOPG) electrodes.

EC-AFM of BF BP-C(HOPG) *vs.* Li/Li^+

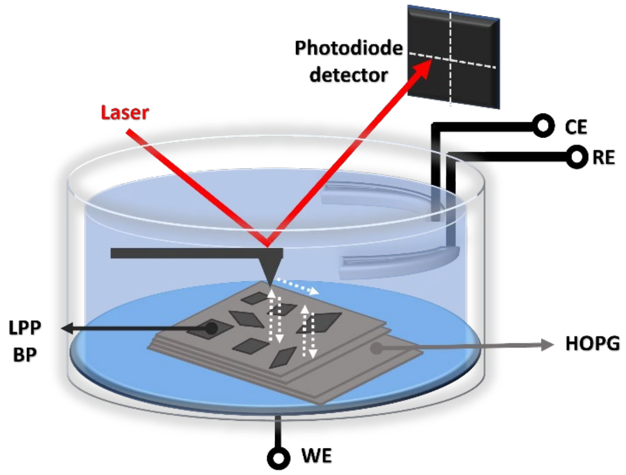

Figure S3: Schematic of the open-topped EC-AFM cell showing the BF BP-C(HOPG) working electrode and Li foil wrapped Ni wire reference plus counter electrodes respectively, immersed in electrolyte. The cell is housed inside an Ar glovebox, enabling liquid imaging with commercial battery electrolytes.

Optical Microscopy Imaging of BF BP-C(HOPG) Electrodes

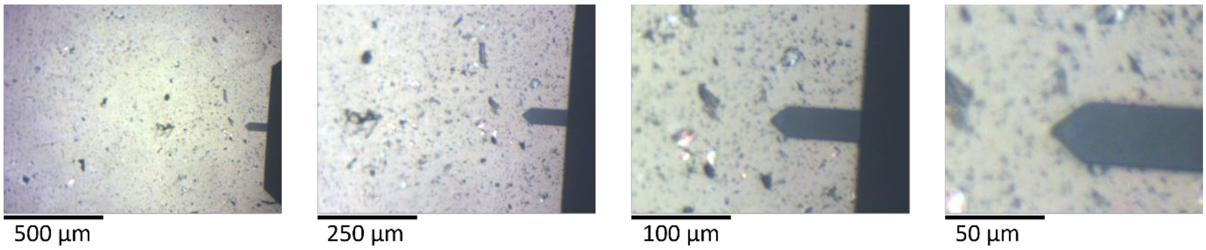

Figure S4: Optical microscopy images of the BF BP-C(HOPG) electrodes in the EC-AFM cell, immersed in 1M $LiPF_6$ and at OCV. Scale bars are shown at the bottom left of each image.

EC-AFM Imaging of the Lithiation Mechanism of BF BP-C(HOPG).

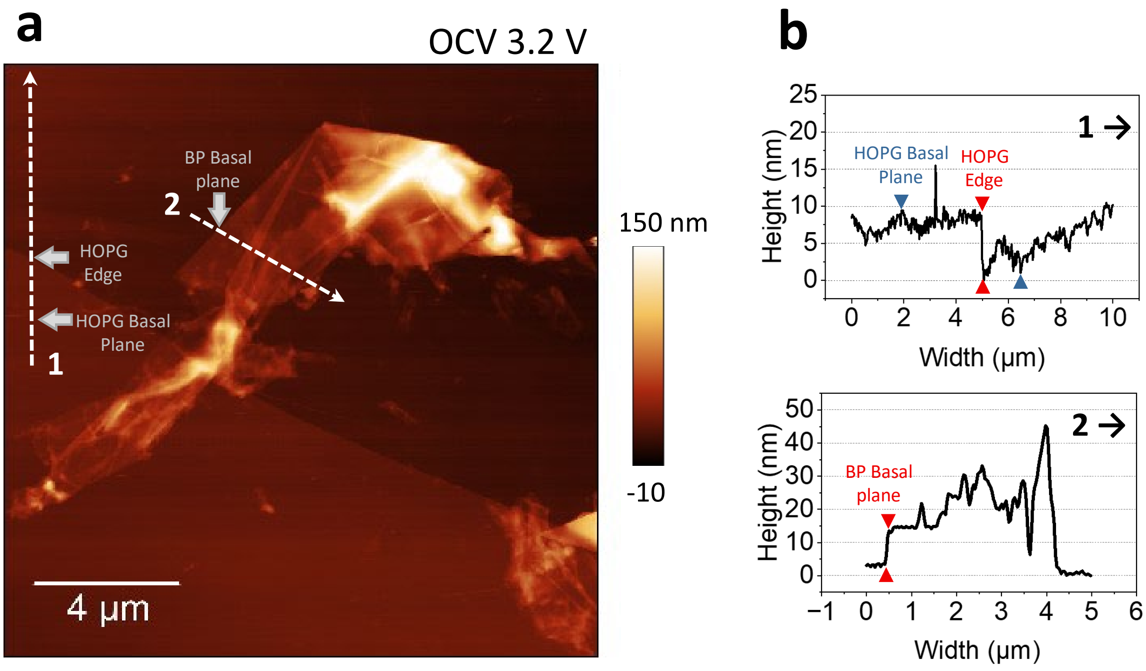

Figure S5: (a) EC-AFM Image of the surface of the BF BP-C(HOPG) anode in the electrochemical cell with 1M $LiPF_6$ EC/DEC electrolyte at OCV, captured across a 15 x 15 μm^2 area. The z-scale bar is shown to the right of image. (b) Height profiles extracted from line 1 and 2 marked in (a), with the direction of the arrow indicating the direction.

Cyclic Voltammetry of BP-C Electrodes Collected in Coin Cells *vs.* EC-AFM Cell.

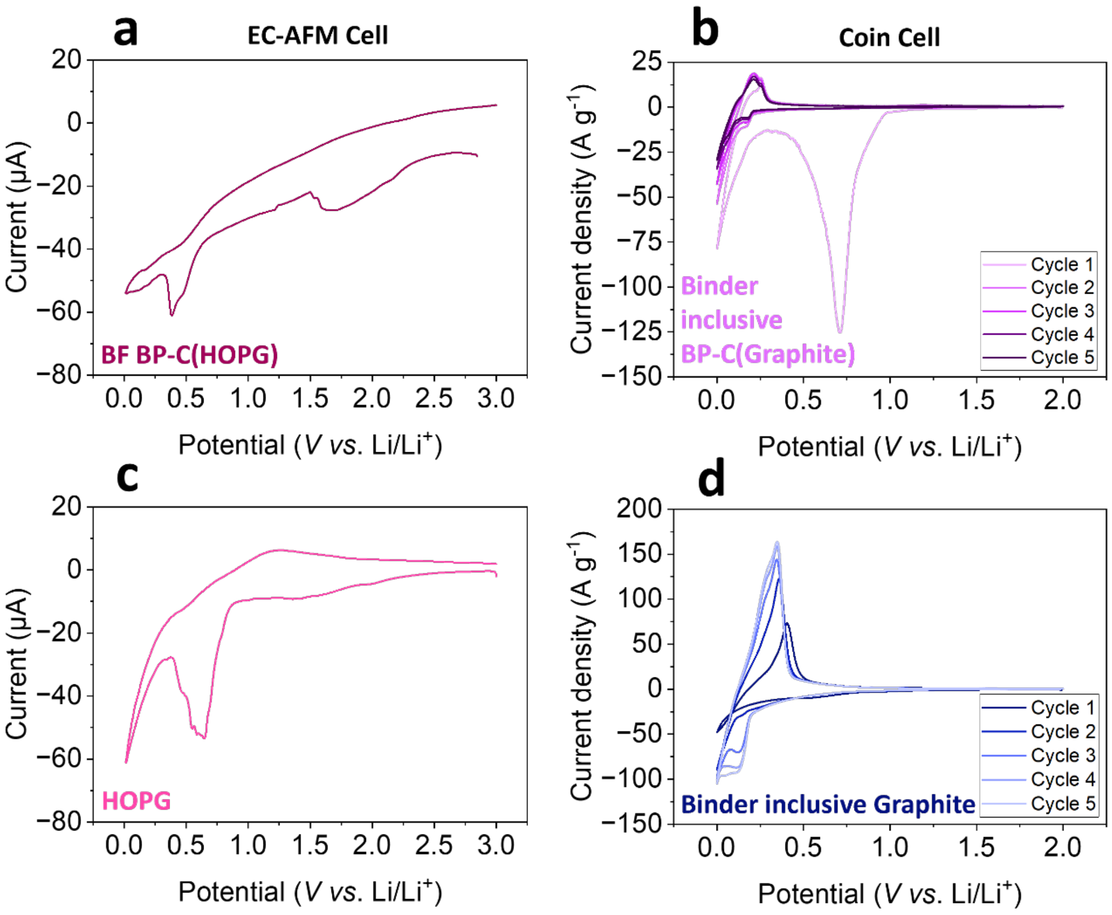

Figure S6: CV comparison of BP-C and graphite cells *vs.* Li metal (a) binder free BP-C(HOPG) electrode collected in the EC-AFM cell (b) binder inclusive BP-C(graphite) electrode collected in a coin cell (c) bare HOPG electrode collected in the EC-AFM cell (d) binder inclusive BP-C(graphite) electrode collected in a coin cell. (a & c) were collected at 0.5 mV s^{-1}; (b & d) were collected at 0.1 mV s^{-1}.

EC-AFM Imaging of the Lithiation Mechanism of the BF BP-C(HOPG) Electrodes

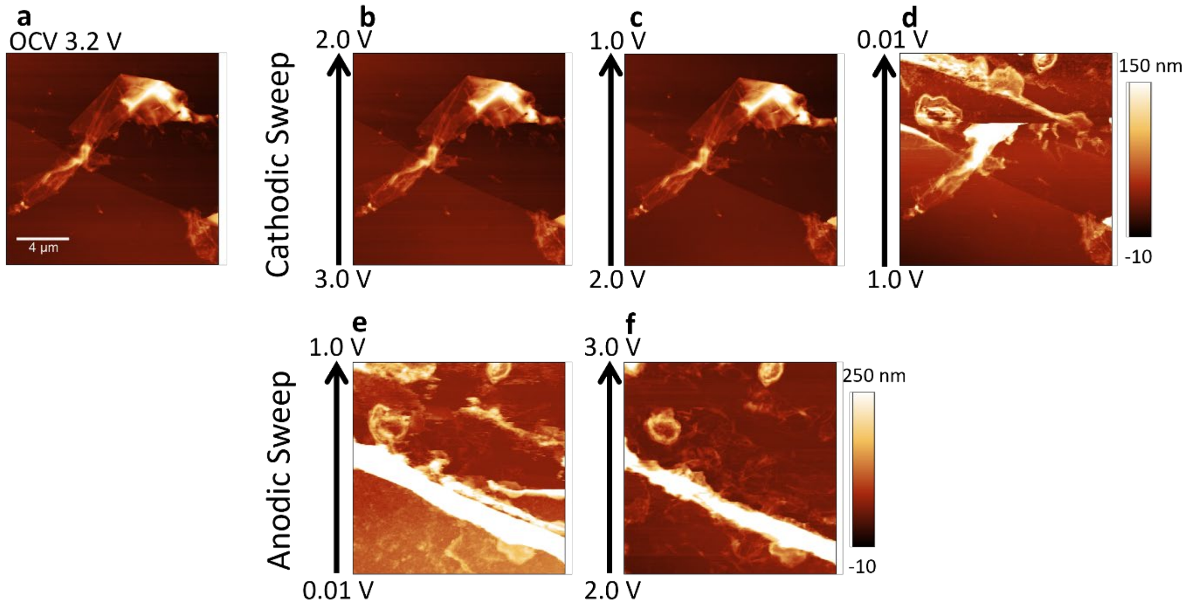

Figure S7: EC-AFM Images of the surface of the BP-C(HOPG) electrode in the electrochemical cell with 1M $LiPF_6$ EC/DEC electrolyte at OCV, inset shows the scale of the image. (a) at OCV (b-f) captured under electrochemical control in between 3 — 0.01 V *vs.* Li/Li^+ at 0.5 mV s^{-1}. All EC-AFM images captured across 15 x 15 µm area. All z-height scale bars to the right of each row.

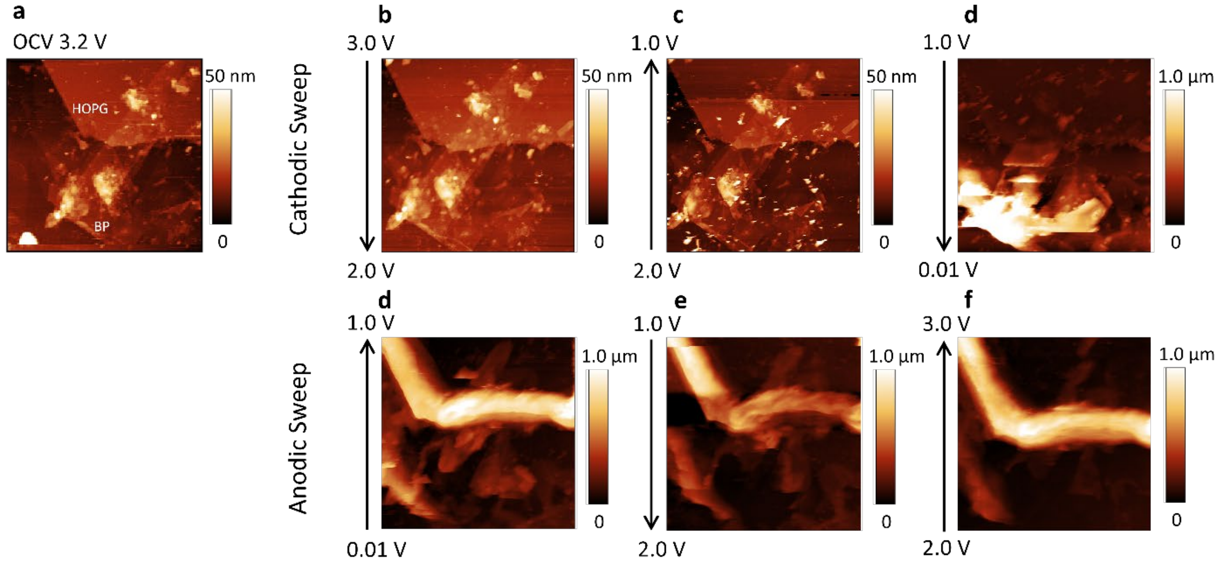

Figure S8: EC-AFM images of the surface of the BP-C(HOPG) electrode *vs.* Li metal in the electrochemical cell with 1M $LiPF_6$ EC/DEC electrolyte. (a) At OCV (b-f) under electrochemical control between 3 — 0.01 V *vs.* Li/Li^+ at 0.5 mV s^{-1}. All EC-AFM images were captured across 6 x 6 µm^2 area, with their corresponding z-height scale reported to the right of the respective image.

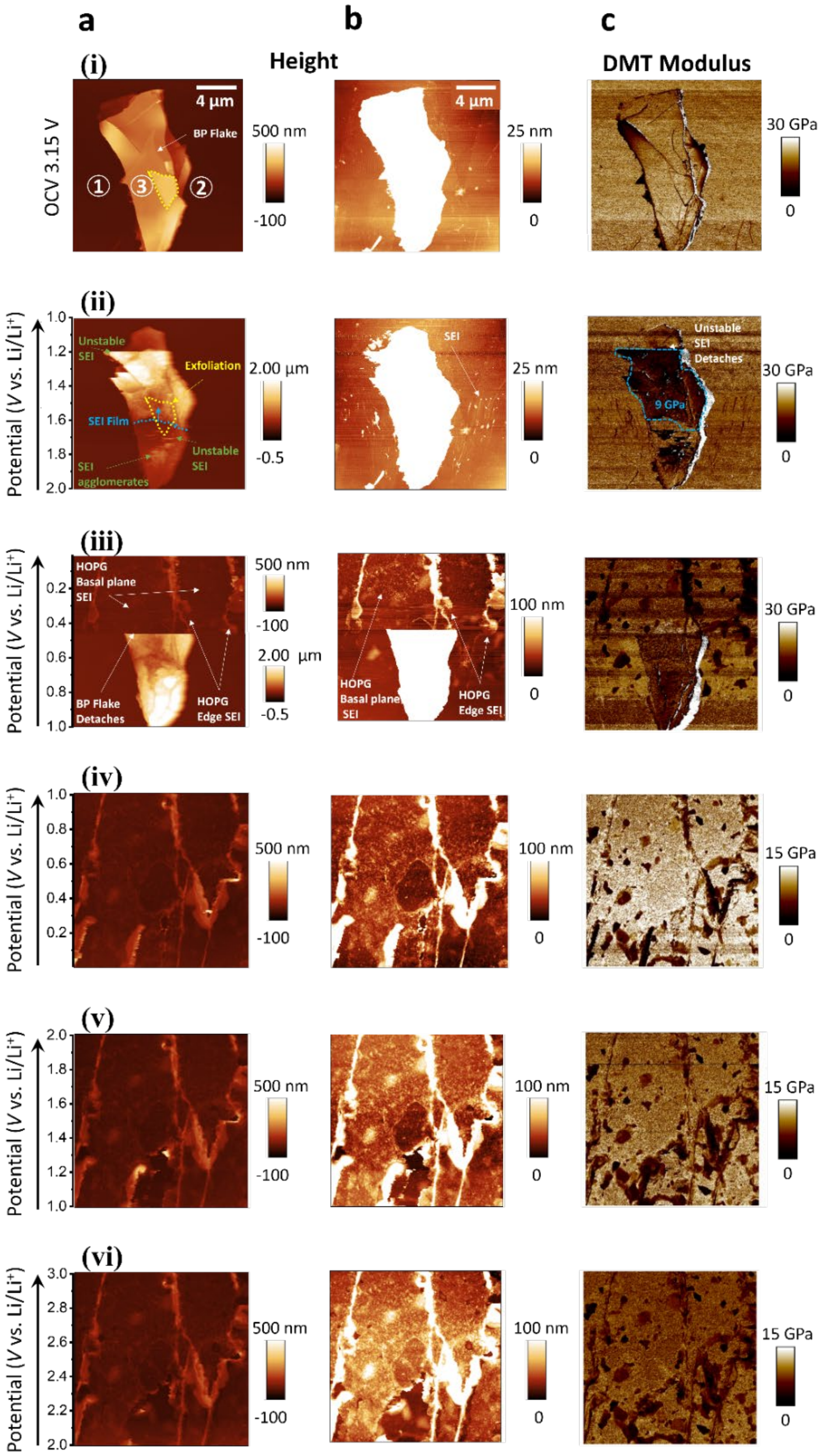

Figure S9: EC-AFM imaging with DMT modulus maps of a binder free BP-C(HOPG) electrode collected whilst under electrochemical control in the range 3 — 0.01 V *vs.* Li/Li$^+$ at 0.5 mV s^{-1} in 1M $LiPF_6$ EC/DEC electrolyte. This Figure accompanies Figure 4. (a) AFM height images (b) AFM height images with adjusted height scale to highlight the topography of the HOPG step edges and associated SEI, and (c) DMT modulus maps. The voltage *vs.* Li/Li$^+$ of each image series is as follows: (i) OCV, (ii) 2.0-1.0 V during discharge, (iii) 1.0-0.01 V during discharge, (iv) 0.01-1.0 V during charge, (v) 1.0-2.0 V during charge, (vi) 2.0-3.0 V during charge. All EC-AFM images were captured across 20 x 20 µm^2 area, and voltages plotted vs. Li/Li$^+$. The black arrow corresponds to the AFM scan direction. All maps are reported with the z/modulus scalebar to the right of the respective image, and x-y scalebar inset in (ai) and (bi).

EC-AFM: Measuring BP-C(HOPG) Feature Heights

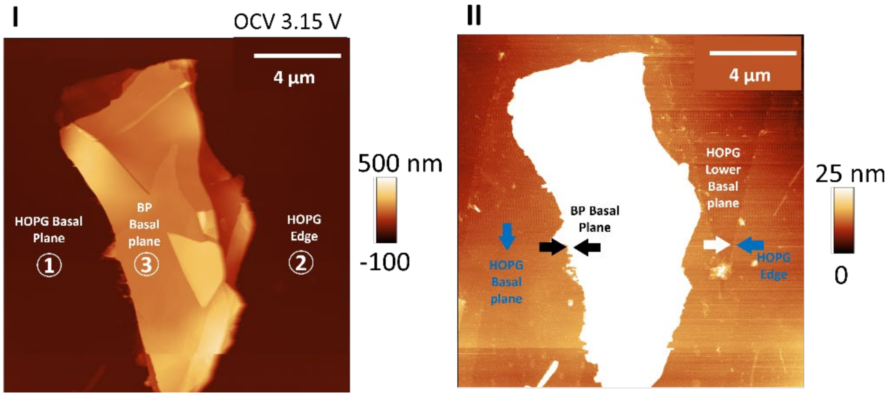

Figure S10: Englarged EC-AFM height images at OCV reproduced from Figure S9ai & bi highlighting the HOPG basal plane (point 1), the HOPG step edge (point 2) and the BP basal plane (point 3).

The heights were measured relative to the normalised lower basal plane as exemplified in Figure S10. Points 1 – 3 labelled in Figure S10 (i) correspond to HOPG basal plane, HOPG edge and BP basal plane, respectively. The HOPG edge and HOPG basal plane (indicated by the blue arrows in Figure S10 (ii)) were measured relative to lower HOPG plane (white arrow). The BP basal plane (black arrow) was also measured to the lower adjacent HOPG basal plane (black arrow). This measurement was replicated across each EC-AFM images presented in Figure S9, whilst under electrochemical control. Given that the voltage is not constant across the line scan, the height and DMT modulus is plotted as a function of the voltage range in which the AFM measurement was taken (Figure 4c (i) and (ii) respectively).

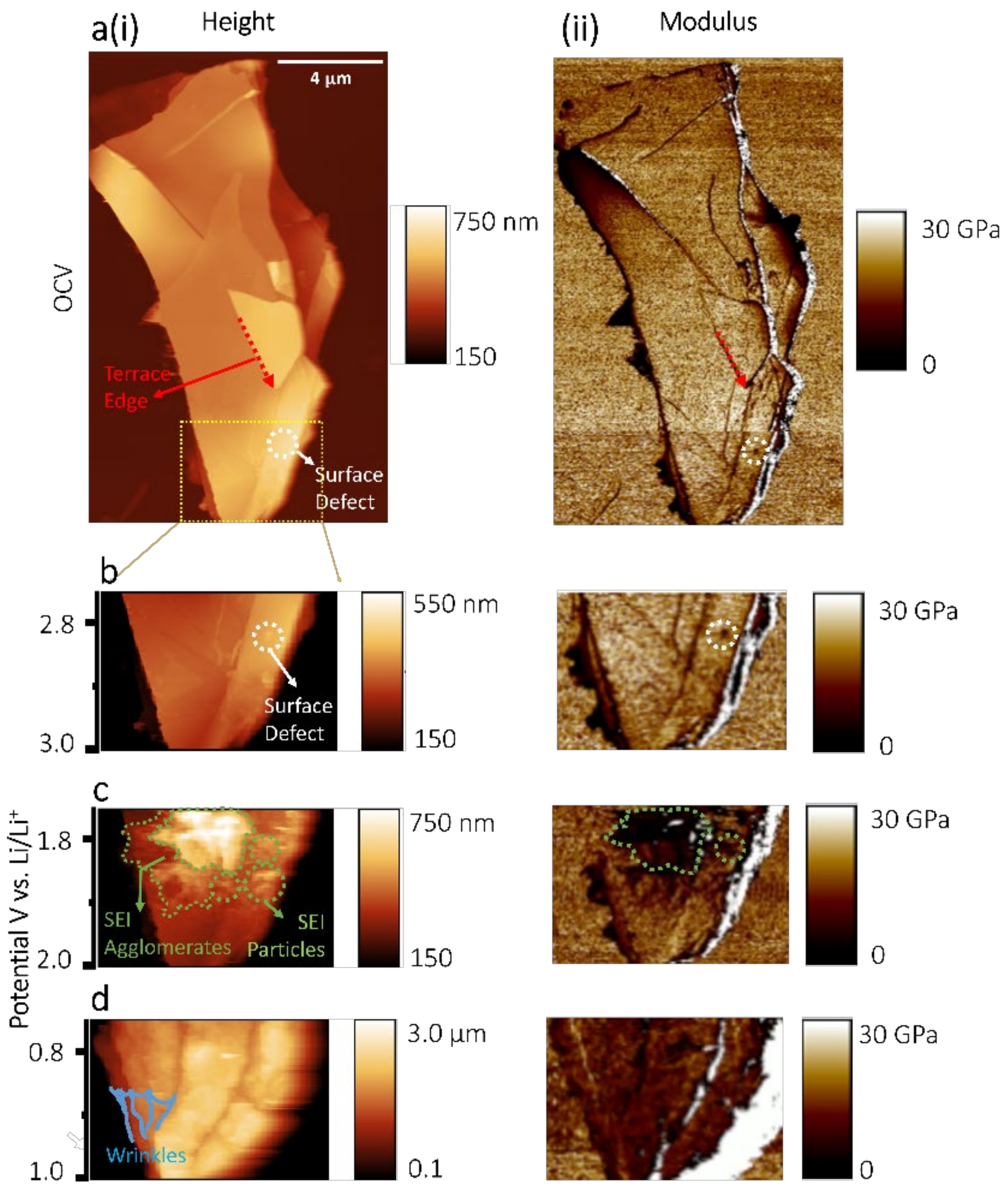

Figure S11: EC-AFM morphology (i) and DMT modulus (ii) maps of the binder free BP-C(HOPG) electrode (a) at OCV, across 18.67 x 11.95 µm area, inset shows the scale bar, (b-d) EC-AFM height and modulus maps of the magnified BP (6.45 x 4.25.µm area) whilst under electrochemical control, taken across voltage range specified to the left of each image. All images were captured in 1M $LiPF_6$, voltages plotted *vs.* Li/Li^+, and all map scale bars are shown to the right of each image.

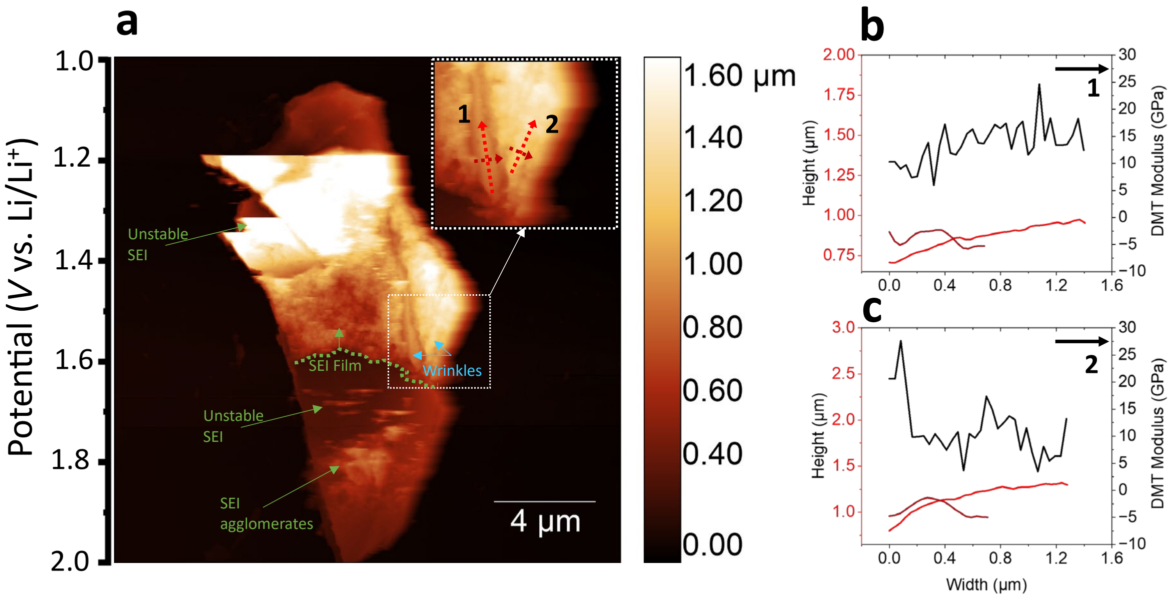

Figure S12: (a) Operando EC-AFM morphology map of the binder free BP-C(HOPG) anode, in 1M $LiPF_6$ EC/DEC, captured during the cathodic sweep 2.0 – 1.0 V. Inset shows the scale bar of the image, and z-scale height shown to the right. (b) Plot of the height, width and DMT modulus line scan across wrinkle 1, and wrinkle 2 (c), marked by the red arrows in (a).

XPS: Characterizing the Chemical and Structural Composition of the SEI/BP-C Interface

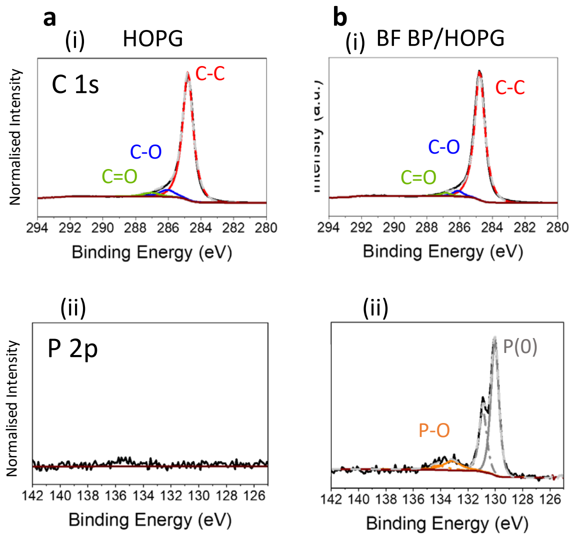

Figure S13: High resolution XPS spectra of C 1s (i) and P 2p (ii) for the (a) Pristine HOPG (b) and Pristine BF BP-C(HOPG).

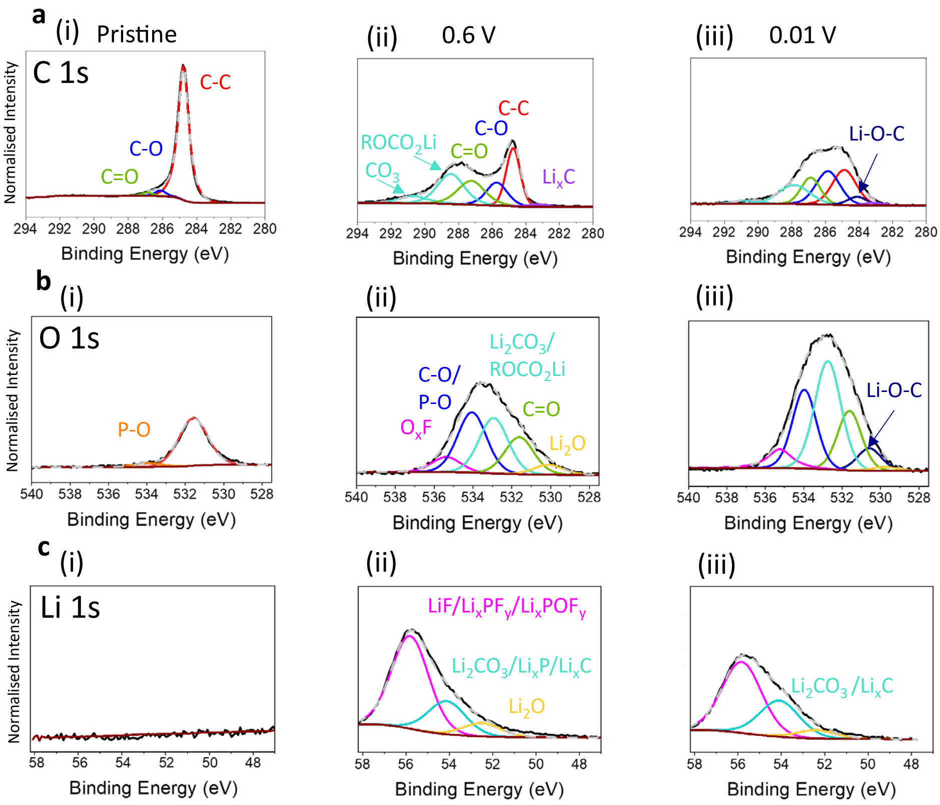

Figure S14: High resolution XPS C 1s (a), O 1s (b), and Li 1s spectra for the (i) pristine binder free BP-C(HOPG) electrode (ii) binder free BP-C(HOPG) electrode cycled in the range 2.5 – 0.6 V and (iii) cycled in the range 2.5 – 0.01 V.

Figure S14 shows the C 1s, O 1s and Li 1s spectra for the composite BF BP-C(HOPG) electrodes in the pristine form (Figure S14a-c (i)), cycled to 0.6 V (Figure S14a-c (ii)) and 0.01 V (Figure S14a-c (iii)) respectively. The C 1s spectra for the pristine BP-C(HOPG) electrodes shows peaks at 284.8 (C–C), 286.0 (C–O), and 287.3 (C=O) eV. In the cycled electrodes new peaks at 288.4 (Semi-carbonates), and 290.7 (CO_3) which are typical of carbonate like species that could be assigned to inorganic species such as Li_2CO_3, $ROCO_2Li$ and organic species, such as polycarbonates, generated by the reduction of solvents in SEI. [1] An additional extremely low binding energy of 283.2 eV found was ascribed to C in the lithiated Li_x–C electrode. [2]

The O 1s spectra presented in Figure S14b (i-iii), are more complicated to deconvolute, due to the presence of many overlapping bonding states expected in all samples from adventitious carbon-oxygen environments in addition to those arising from other bonding environments

within the sample. In the Pristine sample Figure S14b (i), a minimum of two peaks were fitted, the O1s peak centred at ~534 eV, were assigned to P-O bonding environments, as has been reported in other studies.[3] For the cycled electrodes, new peaks presented themselves, and a minimum of 6 peaks were assigned to known SEI species that have been widely reported in literature which are reported in Table 1. These correspond to; Li−O at 529.6 eV, C=O at 531.5 eV, semi-carbonates and carbonates at 532.7 eV, C–O and P–O overlapping at 534.0 eV, and O–F at 535.2 eV.

The Li 1s XPS peak, again is difficult to deconvolute and a minimum of 3 peaks have been defined at 52.3 eV, 54.0 eV and 55.8 eV. According to literature, the peak at 52.3 eV is assigned to Li_2O. [4] The broad peak at 54.0 eV likely has overlapping contributions from Li_2CO3, [4] Li_xP, [3] and Li_xC,[5] in the case of the electrode cycled above alloying, 0.6 V, whereas Li_xP was not detected for the electrode cycled below alloying potential, 0.01 V, due to the absence of this peak in the P 2p spectra (Figure 5). Finally, the peak at 55.8 eV likely has overlapping contributions from LiF, Li_xPF_y and/or $Li_xPF_yO_z$. These results further confirm the existence of inorganic compounds with Li–O and Li–F bonds within the SEI matrix, as well as Li reduction products of C and P. Overall, the combined spectra confirm the presence of known components of LIB SEIs from the double-electron reduction process of EC which are carbonate like organic components such as, $ROCO_2Li$ and polycarbonates and inorganic compounds containing such as Li_2O and Li_2CO_3, and salt reduction products such as LiF Li_xPF_y and/or $Li_xPF_yO_z$. [4–8]

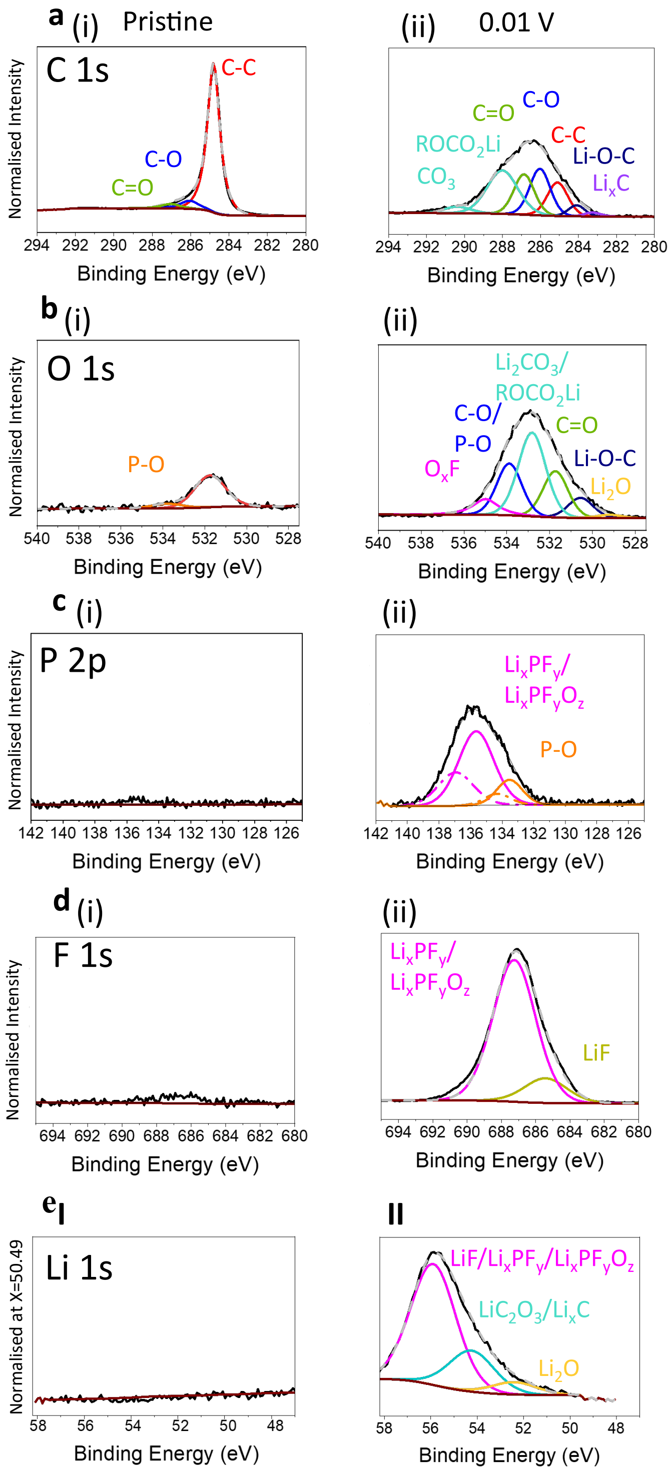

Figure S15: High resolution XPS C 1s (a), O 1s (b), P 2p (c) F 1s (d) and Li 1s (e) spectra the pristine bare HOPG electrode (i), and the bare HOPG electrode cycled in the range 2.5 – 0.01 V .

Table S1: Reported high resolutions XPS spectral binding energies for common LIB SEI components and BP related components.

Material	Hight Resolution XPS spectral Binding Energy				
	O 1s	***C 1s***	***F 1s***	***P 1s***	***Li 1s***
Li_xC		282.0[2,4]	-	-	~54[5]
Li_2O	528.8[5]		-	-	53.7[4]
Li-O-C	530.0[4]	284.0[4]	-	-	55.0[4]
Semi-Carbonates e.g $ROCO_2Li$	532.2[4]	289.0[4]	-	-	
Li_2CO_3	532.5[2,4,5]	290.5[2,4]	-	-	55.2[4]
Li-F			685.5-686.2 [4,5]	-	56. 4[4]
C=O	531.0[4]	287.0[4]	-	-	-
C-O	534.0 [5]	285.0[5]	-	-	-
C-C	-	284.8[4]	-	-	-
Li_xPF_y		-	687.2 [9]	135.5-136.7 [9]	
$Li_xPF_yO_z$	535.2[9]		687.2[9]	135.5-136.7[9]	
Li_xP	-	-	-	128.9[9]	
Phosphate	534 [3]	-	-	134.5[3]	-
P(0)	-	-	-	130	-

REFERENCES

(1) Dedryvère, R.; Martinez, H.; Leroy, S.; Lemordant, D.; Bonhomme, F.; Biensan, P.; Gonbeau, D. Surface Film Formation on Electrodes in a LiCoO2/Graphite Cell: A Step by Step XPS Study. *J Power Sources* **2007**, *174* (2), 462–468.

(2) Högström, K. C.; Malmgren, S.; Hahlin, M.; Rensmo, H.; Thébault, F.; Johansson, P.; Edström, K. The Influence of PMS-Additive on the Electrode/Electrolyte Interfaces in LiFePO4/Graphite Li-Ion Batteries. *Journal of Physical Chemistry C* **2013**, *117* (45), 23476–23486.

(3) Quartarone, E.; Eisenmann, T.; Kuenzel, M.; Tealdi, C.; Marrani, A. G.; Brutti, S.; Callegari, D.; Passerini, S. Towards Advanced Sodium-Ion Batteries: Green, Low-Cost and High-Capacity Anode Compartment Encompassing Phosphorus/Carbon Nanocomposite as the Active Material and Aluminum as the Current Collector. *J Electrochem Soc* **2020**, *167* (8), 7196–7204.

(4) Bar-Tow, D.; Peled, E.; Burstein, L. A Study of Highly Oriented Pyrolytic Graphite as a Model for the Graphite Anode in Li-Ion Batteries. *J Electrochem Soc* **1999**, *146* (3), 824–832.

(5) Kanamura, K.; Shiraishi, S.; Takezawa, H.; Takehara, Z. I. XPS Analysis of the Surface of a Carbon Electrode Intercalated by Lithium Ions. *Chemistry of Materials* **1997**, *9* (8), 1797–1804.

(6) Xu, S. D.; Zhuang, Q. C.; Wang, J.; Xu, Y. Q.; Zhu, Y. B. New Insight into Vinylethylene Carbonate as a Film Forming Additive to Ethylene Carbonate-Based Electrolytes for Lithium-Ion Batteries. *Int J Electrochem Sci* **2013**, *8*, 8058–8076.

(7) Soto, F. A.; Yan, P.; Engelhard, M. H.; Marzouk, A.; Wang, C.; Xu, G.; Chen, Z.; Amine, K.; Liu, J.; Sprenkle, V. L.; El-Mellouhi, F.; Balbuena, P. B.; Li, X. Tuning the

Solid Electrolyte Interphase for Selective Li- and Na-Ion Storage in Hard Carbon. *Advanced Materials* **2017**, *28* (18), 1606860.

(8) Heiskanen, S. K.; Kim, J.; Lucht, B. L. Generation and Evolution of the Solid Electrolyte Interphase of Lithium-Ion Batteries. *Joule* **2019**, *3* (10), 2322–2333.

(9) Dahbi, M.; Yabuuchi, N.; Fukunishi, M.; Kubota, K.; Chihara, K.; Tokiwa, K.; Yu, X. F.; Ushiyama, H.; Yamashita, K.; Son, J. Y.; Cui, Y. T.; Oji, H.; Komaba, S. Black Phosphorus as a High-Capacity, High-Capability Negative Electrode for Sodium-Ion Batteries: Investigation of the Electrode/Electrolyte Interface. *Chemistry of Materials* **2016**, *28* (6), 1625–1635.
